# Modeling microplastic dynamics in riverine systems: fate and transport analysis

**DOI:** 10.1007/s11356-025-36875-6

**Published:** 2025-08-22

**Authors:** Nerea Portillo De Arbeloa, Alessandra Marzadri

**Affiliations:** University of Trento Department of Civil Environmental and Mechanical Engineering: Universita degli Studi di Trento Dipartimento di Ingegneria Civile Ambientale e Meccanica ITALY, Trento, Italy

**Keywords:** Microplastics, River networks, Transport processes, Modeling approach

## Abstract

The escalation of plastic production and its ubiquitous application across numerous sectors have led to increased plastic waste, contributing to the widespread occurrence of microplastics (MP) across different ecosystems. Due to their durability and resistance to breakdown, these particles persist and accumulate in the environment, threatening both ecological and human health. Rivers serve as critical conduits that link terrestrial and marine environments, thus playing a pivotal role in the distribution of MP. Consequently, one of the key research priorities is focused on understanding the primary processes that controls the transport of MP in riverine environments. Here, we propose an integrated version of a previously published model that solves the classical advection-dispersion-reaction equation (ADRE) by mimicking the removal/resuspension of MP as reactive terms. The proposed framework integrates inputs of MP from human activities and assesses transport and deposition processes—including sedimentation, burial, resuspension, and removal along riverbanks—tailored to the specific hydro-geomorphological characteristics of river segments. The model was applied in a real river network, the Tame River (UK), to evaluate four distinct MP composition scenarios: a high-fiber scenario, a high-fragment scenario, a high-pellet scenario, and an averaged composition scenario derived from literature. These scenarios represent realistic variations in particle morphology and density, allowing an assessment of how particle-specific properties influence transport and retention. The results highlight the dominant role of bank retention and sedimentation in MP transport, demonstrating that depositional processes significantly contribute to the long-term retention of MP in river networks. This tool is instrumental in enhancing our understanding of MP pollution in river systems and proposing effective mitigation strategies.

**Supplementary Information:**

The online version contains supplementary material available at 10.1007/s11356-025-36875-6.

## Introduction

The widespread distribution of microplastics (MP) in aquatic environments is a critical environmental issue that threatens biodiversity, water quality, and ecosystem health (Nizzetto et al. 2016a; Drummond et al. 2022). MP, defined as plastic particles with size ranging between $1\, \mu m$ and 5 mm (Bermúdez and Swarzenski 2021), originate from a variety of sources, including industrial processes, household waste, and the breakdown of larger plastic debris (Tirkey and Upadhyay 2021). Once introduced into freshwater systems (i.e., streams and rivers and their connected surface and subsurface environments), MP go through several physical, chemical, and biological processes that control their transport, fate, and potential impacts on riverine environments (Ali et al. 2024).

Physical interactions play a significant role in the behavior of MP within aquatic systems. Key among these interactions is advection, the process by which MP particles are transported downstream by the bulk movement of water. This process is also affected by the specific characteristics of MP, such as size and density, which influence their settling in the water column or on the riverbed, leading to further interactions with the benthic and hyporheic environments (Drummond et al. 2022; Guo et al. 2024). These interactions include transformation processes such as agglomeration, aggregation and degradation, and transport processes, including resuspension, retention, or burial into the streambed sediments (i.e., mainly benthic and hyporheic zone) (Kooi et al. 2018; Xia et al. 2023; Teitelbaum et al. 2022; Peleg et al. 2024). For the purpose of better predicting MP trajectories and effects across diverse ecosystems, the research community has undertaken a variety of efforts, including data collection and analysis, experimental investigations, and simulation studies (Waldschläger et al. 2022; Uzun et al. 2022). Specifically, mathematical models have been developed to delineate the behavior and interactions of MP within various fluvial ecosystem compartments, such as streams, sediments, and banks, under different flow conditions and scenarios. MP mathematical models can be categorized into several types, as outlined by Uzun et al. (2022), including hydrodynamic, statistical, mass-balanced, and process-based models.

In river networks, the application of models integrating hydrodynamics and process-based methodologies was previously used to characterize the fate of nutrients and greenhouse gas emissions (see for example some of the models present in literature (Seitzinger et al. 2010; Maavara et al. 2019; Yao et al. 2020; Marzadri et al. 2021)). These models offer a comprehensive approach to understanding the complex interactions within river systems, capturing the intricate dynamics of flow and incorporating detailed analyses of various geomorphological, hydrological and biogeochemical processes. Similar frameworks can be used also for analyzing the transport of MP. For example, Besseling et al. (2017), building upon the foundational work of Quik et al. (2015), delved into the fate and transport of engineered nanoparticles and microplastics by assessing various scenarios. Their analysis encompassed advective transport, homo- and hetero-aggregation, sedimentation-resuspension dynamics, polymer degradation, and the effects of biofilm presence and burial processes by evaluating a series of different scenarios. Notably, one scenario overlooked spatial heterogeneity, the variability of conditions across different locations, and the diversity in particle characteristics. This omission led to a significant decrease in retention values, underscoring the critical need for detailed characterization of the particles shape. Their model was specifically designed for particles with nearly perfect shapes, highlighting a significant limitation due to the omission of how particle shape influences retention. Additionally, while the model primarily concentrates on nanoparticles, it has not been validated for MP, thereby introducing a further area for research exploration.

In their study of MP pollution dynamics within Swiss rivers, Mennekes and Nowack (2023) employed a methodological approach to assess how various polymers were retained by lakes and rivers. They calculated sedimentation rates utilizing a negative compound interest model, which relied on travel times and sedimentation rates from literature, but overlooked the influence of particle shape on sedimentation behavior. For estimating the accumulation or burial rate, they assumed that $10\%$ of the particles in sediments would undergo burial, aligning with values reported in prior studies (Domercq et al. 2022; Besseling et al. 2017). However, this method did not account for the effect of different stream morphology (i.e., dune, ripples) and specific particle characteristics that could significantly affect sedimentation and accumulation processes, potentially limiting the accuracy of their retention estimates. Furthermore, Nizzetto et al. (2016b) estimated the settling velocity and entertainment of MP through mathematical formulations that incorporate the density and median diameter of the MP particles. Yet, they acknowledged that their approach does not fully represent the behavior of MP due to the variation in shape and density. Portillo De Arbeloa and Marzadri (2024) introduced a model based on the advection dispersion equation, incorporating both anthropogenic MP loads and river network characteristics. This model assumes equilibrium within the transport processes. While the outcomes were able to represent a possible scenario of transport, they tend to overestimate MP concentrations within river systems where removal and resuspension processes play non-negligible effect, indicating a potential area for enhancement.

MP sedimentation dynamics driven by the characteristics of MP particles, such as shape, density, and how they behave under various flow conditions, remains a topic in need of more comprehensive research. Another process that has not been taken into consideration to date is the potential role of lateral cavities (i.e., and their capacity to influence MP retention). Lateral cavities are macro-roughness regions along the banks of a river where there is a change of water flow dynamics, sediment deposition, and nutrient cycling (Juez et al. 2018). They enhance biodiversity and aid in biogeochemical cycling by capturing suspended particles (Jackson 2010). This characteristic indicates that lateral cavities could significantly impact MP concentrations within aquatic environments. Their ability to collect and retain particulate matter could extend to MP, potentially leading to increased MP accumulation in these areas. In consequence, this could shift the distribution and ecological effects of MP in water bodies.

In this work, we proposed a spatially explicit model that solves the classical advection-dispersion-reaction equation (ADRE) (Van Genuchten 1981), where the reactive component is represented by a first-order decay rate that considers the transport processes such as sedimentation, burial, resuspension, and bank retention. The sedimentation rate is addressed by the settling velocity formulation proposed by Francalanci et al. (2021). The burial processes, which describe the process in how the particles are incorporated into the streambed (Mennekes and Nowack 2023), are estimated using a similar approach to the one proposed by Domercq et al. (2022) represented by ratio between mean downwelling hyporheic flux and its depth. To describe the resuspension process, we have included a method that accounts for a MP resuspension flux once the critical shear stress of the riverbed is exceeded (Quik et al. 2015; Yu et al. 2023; Waldschläger and Schüttrumpf 2019). Finally, to account for the bank retention effect, we introduce a removal rate dependent on the transient storage times that water spent within the lateral zones of the river. Our model was integrated within the framework proposed in Portillo De Arbeloa and Marzadri (2024) and was applied to a sub-catchment of the Tame River (England) (Woodward et al. 2021). To evaluate how particle characteristics influence transport behavior and retention, we implemented four MP composition scenarios: a high-fiber scenario, a high-fragment scenario, a high-pellet scenario, and a scenario based on average values reported in the literature (Akdogan et al. 2023; Liu et al. 2023; Schrank et al. 2022; Yuan et al. 2022; Margenat et al. 2021; Woodward et al. 2021; Han et al. 2020; Mani et al. 2015). These scenarios allowed us to explore the impact of particle morphology and density on MP fate in a real river network. Model outputs provide new insights into the mechanisms governing MP transport and support improved assessments of pollution risk and potential mitigation strategies.

## Methods

**Fig. 1:**
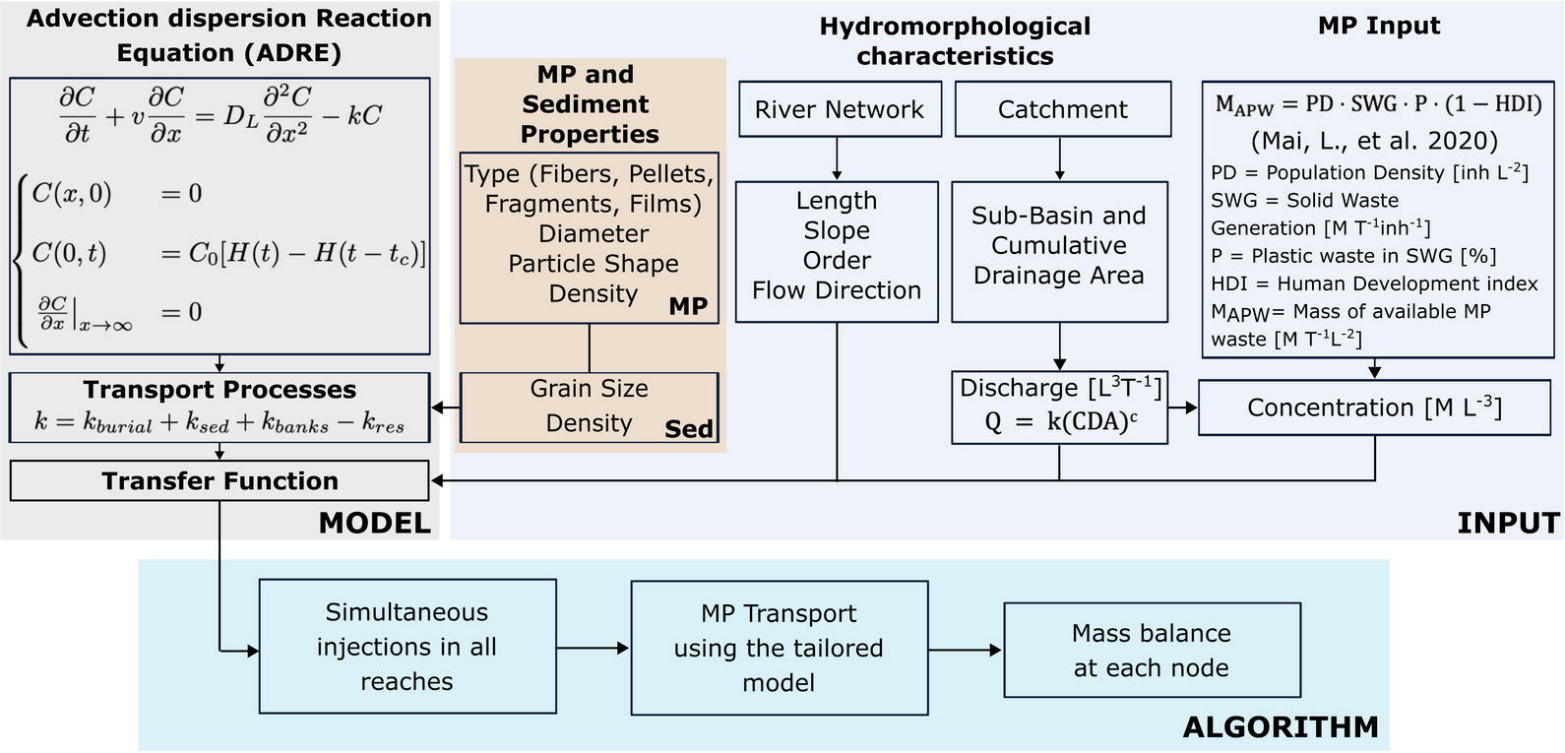
Overview of the modeling procedure. Modified from Portillo De Arbeloa and Marzadri (2024)

In this section, we provide a detailed explanation of the mathematical formulation used to represent the mechanisms involved in the transport and removal of MP. As underlined in the introduction section, we characterize the MP transport by considering the interplay among the main processes: advection, dispersion, burial, resuspension, bank removal, and sedimentation that, according to the properties (i.e., shape, density) of MP particles, controls their movement within the different fluvial ecosystem compartments (i.e., water column, lateral cavities, and streambed sediments). Figure 1 shows an overview of the modeling framework with details on: the required inputs, the equations, and the algorithm used to schematize the transport-transformation processes that controls MP along river networks.

### Mathematical model

The model solves the one-dimensional ADRE along the downstream direction by tailoring the transport of MP through the different river reaches that compose the fluvial networks. At the river network scale, this choice is recommended (respect to two- and three-dimensional models) to estimate possible pollutants fate by reducing the computational time (Launay et al. 2015; Fardadi Shilsar et al. 2023). Within this framework, the MP transport can be represented as follows:

1 $$\begin{aligned} \frac{\partial C}{\partial t} + v \frac{\partial C}{\partial x} = D_{L} \frac{\partial ^2C}{\partial x^2} - kC \end{aligned}$$

where $C \; [ML^{-3}]$ is the MP concentration in the river reach, $t\;[T]$ is the time, $v\;[LT^{-1}]$ is the mean stream velocity, $x \; [L]$ is the longitudinal coordinate along the reach, $D_{L}\;[L^2 T^{-1}]$ is the hydrodynamic dispersion coefficient, and $k\;[T^{-1}]$ is a first-order decay/production rate that account for the removal/resuspension of MP (i.e., is positive in case of prevailing removal, while is negative in case of prevailing resuspension). Our approach follows a Lagrangian framework, where MP transport is modeled as a function of reach-specific hydromorphological characteristics, such as velocity, discharge, and morphology. Within this framework, the MP concentration along both the cross-section and the water column is assumed to be uniform. This assumption is partially supported by recent studies (Matjašič et al. 2023) and balanced by representing the possible interaction with other fluvial compartments, such as lateral banks and streambed sediments, via opportune reaction rates (i.e., sedimentation, burial, resuspension, and bank removal). The river network is structured as a nested system of (i) sub-basins where, according to the population, MP are generated; (ii) nodes which are set at major hydraulic changes (i.e., inlet and confluence nodes), where interactions with lateral and vertical transport processes are accounted for and where the inputs and dilution (in confluence nodes only) of MP occur; and (iii) reaches where transport-transformation processes occurs (see Portillo De Arbeloa and Marzadri 2024 for major details). Among the possible scenarios of pollutant injection, in the present study, we consider the one that assumes MP being injected in inlet and confluence nodes only and not as distributed sources along the reach (Fardadi Shilsar et al. 2023; Fan et al. 2015). This allows the model to capture spatial variability while ensuring that particle movement dynamically reflects the changing river conditions along the entire network.

Within this framework, $D_{L}$ is estimated using Eq. 2 as a function of the kinematic wave celerity $(v_{w}\;[LT^{-1}])$ (Saco and Kumar 2002) (Eq. 3), mean water depth ($h\;[L]$), and reach slope ($S\;[-]$).

2 $$\begin{aligned} D_{L} = \frac{v_{w}\cdot h}{3\cdot S} \end{aligned}$$

3 $$\begin{aligned} v_{w} = \frac{3}{2}v \end{aligned}$$

**Fig. 2:**
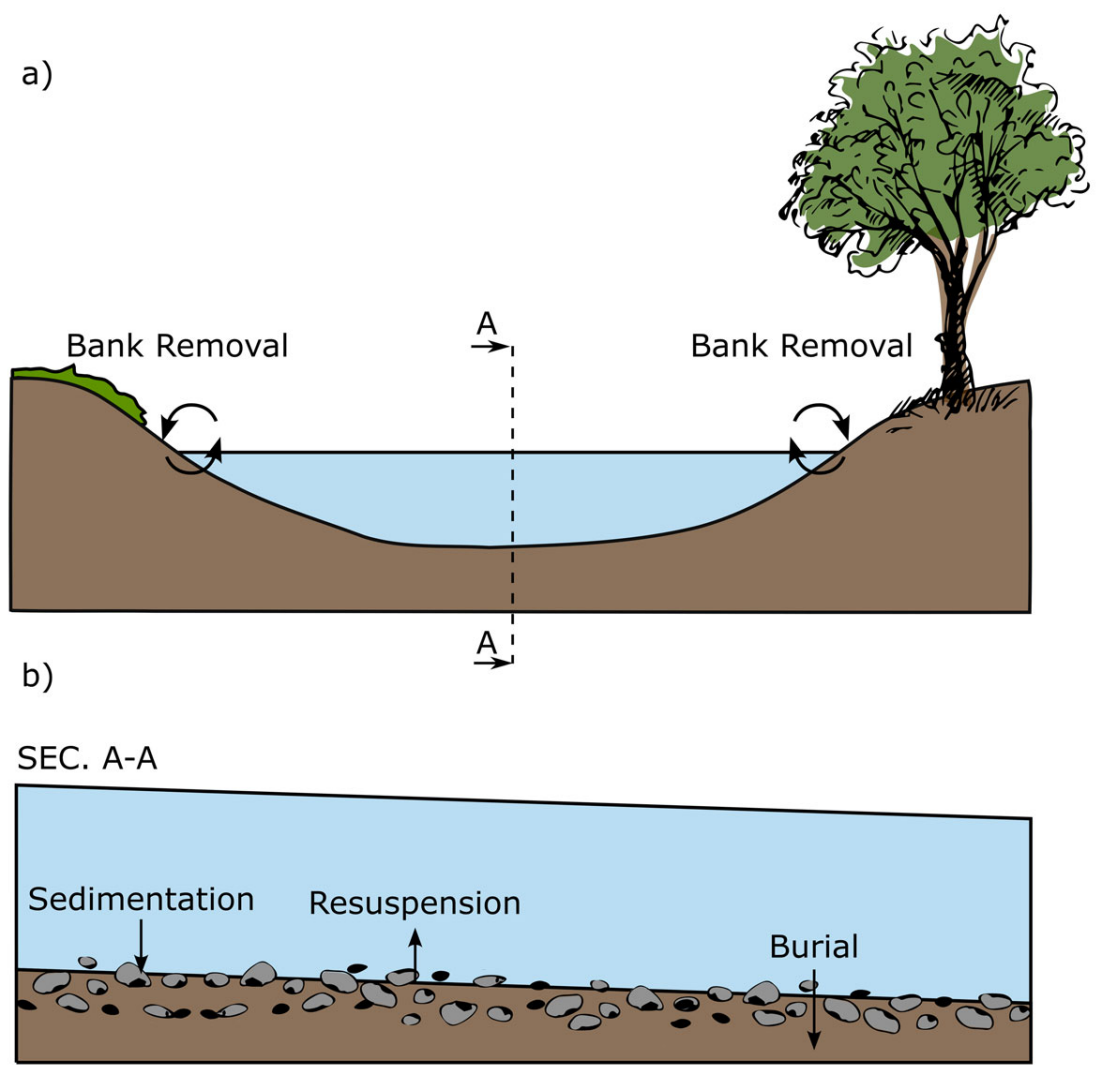
Sketch of the main processes that controls removal-release of MP in fluvial settings. **a** The interaction between surface water and banks through bank removal. **b** The cross-section A:A representing the interaction between surface water and streambed sediments through sedimentation, resuspension, and burial processes

The first-order decay rate, *k* $[T^{-1}]$, can be expressed as the sum of individual decay rate components: the burial rate ($k_{bur}$), the sedimentation rate ($k_{sed}$), the banks retention rate ($k_{banks}$), minus the resuspension rate ($k_{res}$) as shown in Eq. 4. The processes are illustrated in Fig. 2 while their derivation is discussed in “Transport and removal mechanisms.”

4 $$\begin{aligned} k = k_{bur} + k_{sed} + k_{banks} - k_{res} \end{aligned}$$

To approach the problem, we first define the initial and boundary conditions for the concentration of MP within the studied section. Initially (at time $t=0$), the concentration of MP is assumed to be zero throughout the river network, as specified by Eq. 5a. For the boundary conditions, the release of MP is depicted as a constant pulse for a given time period Eq. 5c, and the reach is assumed to be sufficiently long such that its end has a negligible effect on the concentration gradient as *x* approaches infinity Eq. 5b. Under these conditions and following Van Genuchten (1981)’s method, we are able to derive the analytical solution of the transport problem reported in Eq. 1, exploring different scenarios of initial and boundary setups as detailed in the Supporting Information (SI, Section S5).

### Transport and removal mechanisms

#### Burial

For the estimation of the burial rate ($k_{bur}$), defined as the rate at which MP particles become entrapped and accumulate within the riverbed sediments (i.e., within the hyporheic zone, HZ) over time along a given reach, we propose that, under average flow conditions, the volume of MP particles that penetrate into HZ equates to the volume of water that exits from it (i.e., downwelling and upwelling fluxes, generated by the pumping mechanism through the uneven distribution of pressure at the water-sediment interface, are in balance Elliott and Brooks 1997; Marzadri et al. 2010). Consequently, the burial rate can be calculated as the ratio between the mean hyporheic downwelling flux ($\overline{q_{HZ}}$, [$LT^{-1}$]) and the hyporheic zone mean depth ($Y_{HZ}$, [*L*]) as follows:

6 $$\begin{aligned} k_{bur} = \frac{\overline{q_{HZ}}}{Y_{hz}} \end{aligned}$$

The determination of $Y_{HZ}$ is significantly influenced by the morphology of the streambed. For instance, in stream settings characterized by dune morphology, the depth is typically approximated as a function of the average flow depth ($Y_0,\; [L]$) (Marzadri et al. 2016). In contrast, for pool-riffle morphology, the hyporheic zone depth is estimated to be comparable to the channel width (*W*, [*L*]) (Marzadri et al. 2010).

Furthermore, the $\overline{q_{HZ}}$, which is contingent upon the streambed morphology, can be determined using specific equations. For streambeds with dune morphology, Eq. 7a is applicable (Tonina 2012), while for pool-riffle morphology, Eq. 7b should be used (Tonina 2012):

7a $$\begin{aligned} \overline{q_{\textrm{HZ}_\textrm{D}}} = \frac{2 \cdot K_H \cdot h_m}{L_{\textrm{bed}}} \cdot \tanh (2\pi ) \end{aligned}$$

7b $$\begin{aligned} \overline{q_{\textrm{HZ}_\textrm{PR}}} = \frac{41.108 \cdot Y_s^{-0.732} \cdot K_H \sqrt{S} \cdot n}{C_z} \end{aligned}$$

where $K_H\;[LT^{-1}]$ denotes the hydraulic conductivity of the streambed sediments, $h_m\;[L]$ represents the amplitude of the water depth oscillation (Shen et al. 1990), $L_{bed}\;[L]$ is the length of the bedform (Eqs. S3 and S7), $Y_S$ indicates the dimensionless bedform height (through the formulation proposed by Ikeda (1982), as reported in Eq. S4), $S\;[L]$ the stream slope, $n\;[-]$ the streambed porosity, and $C_z\;[-]$ the dimensionless Chezy coefficient (refer to Eq. 8).

8 $$\begin{aligned} C_z = 6 + 2.5 \ln \left( \frac{1}{2.5d_s}\right) \end{aligned}$$

where $d_s\,[-]$ (i.e., the ratio between the mean grain size and the mean flow depth) is the grain relative submergence.

The methodology used to classify the morphology type can be found in SI Section S1.

#### Sedimentation

In the context of particles settling in water, Stokes’ law (Stokes 1851) is commonly used to describe the settling of a spherical particle in an infinite calm fluid. Beyond the Stokes regime (i.e., when the Reynolds number, $Re\;>\;1$), inertial forces start to become important in controlling the drag coefficient which is influenced also by shape, roundness, and surface texture, among others. Dietrich (1982) propose a formulation able to take into account of these effects in controlling the settling velocity of particles, therefore more suitable also to characterize the possible accumulation of MP on the riverbed. Starting from this pioneering work, different approaches are proposed in literature to estimate and parameterize the drag coefficient according to different materials and flow conditions (Ferguson and Church 2004; Khatmullina and Isachenko 2017). Recently, Waldschläger and Schüttrumpf (2019) and Francalanci et al. (2021) estimated expressions for the sedimentation rate analyzing specifically the behavior of MP. Here, we use the formulation proposed by Francalanci et al. (2021) to evaluate the settling velocity ($v_s\;[LT^{-1}]$) and the length of the path a MP particle follows to settle (which depend on the mean flow depth $Y_0$ and the length of the reach *x*). By accounting for the diagonal travel distance along the river, we are incorporating the influence of river reach length on the settling of MP into the riverbed. Generally, the length of the river reach, denoted as *x* above, plays a crucial role in determining the likelihood of contaminant settlement. Longer reaches increase the travel time of the pollutant, which is influenced by the river’s hydraulic conditions, thereby potentially increasing deposition rates (Bottacin-Busolin et al. 2011; Thonon et al. 2007). This simplified formulation does not account for local hydraulic variability, turbulence, or lateral storage effects and should be interpreted as an idealized representation of the settling path in flowing water. Therefore, the sedimentation rate is expressed as follows:

9 $$\begin{aligned} k_{sed} = \frac{v_s}{\sqrt{Y_{0}^{2} + x^2}} \end{aligned}$$

with:

10 $$\begin{aligned} v_s = \frac{g\;R\;D^2}{C_1 v + (0.75\;C_2\;g\;R\;D^3)^{0.5}} \end{aligned}$$

where the parameters *R*, *g*, *v*, and *D* represent the relative density [−], gravitational acceleration [$LT^{-2}$], kinematic viscosity of water [$L^2T^{-1}$], and particle diameter [*L*], respectively. Constants $C_1$ and $C_2$ are coefficients dependent on the shape of the particle (see Eqs. 11 and 12), where *E* corresponds to the grain shape factor (Janke 1966) estimated using Eq. 13; *a*, *b*, and *c* denote the length of longest, intermediate, and shortest axis of the particle, and *csf* is the Corey shape factor defined as $c/\sqrt{a\cdot b}$ that goes from $\approx$ 0 to 1, with values closer to 0 corresponding to a disk or a 2D plate, while 1 represents a perfectly round sphere.

11 $$\begin{aligned} C_1 = 18 E^{-0.38} \end{aligned}$$

12 $$\begin{aligned} C_2 = 0.3708 csf^{-0.1602} \end{aligned}$$

13 $$\begin{aligned} E = a\cdot {\left( \frac{a^2 + b^2 + c^2}{3}\right) }^{-0.5} \end{aligned}$$

The relative density is defined according to Eq. 14, wherein $\rho _w \; [ML^{-3}]$ denotes the density of water and $\rho _p \;[ML^{-3}]$ represents the density of plastic which varies based on the MP particle type.

14 $$\begin{aligned} R = \frac{\rho _p - \rho _w}{\rho _w} \end{aligned}$$

#### Bank retention

River banks, representing the transitional boundary between aquatic and terrestrial environments, play a pivotal role in the dynamics of river ecosystems, in the erosion-sedimentation processes, and in controlling water quality (Domínguez et al. 2016). These buffer areas (comprising parafluvial areas, river bends, groynes, etc.), by trapping and releasing part of the stream water, are often referred as surface transient storage (STS) zones characterized by a reduction of water velocities, an increase of the residence time, and the potential to retain and possibly store part of the river contaminants (Stewart et al. 2011; Jung et al. 2024). Consequently, their role could be crucial also for understanding the transport of MP within river systems.

Discharge, influencing the quantity of contaminants entering the STS zones and the duration they remain there, is the primary driver that shapes the dynamics in these areas (Natho et al. 2020). Higher discharge rates typically result in more contaminants traveling along the main channel, thus reducing the amount entering the STS (Przyborowski et al. 2024; Jung et al. 2024). The effect of river banks on contaminants is largely dependent on the residence time within these transient storage zones. To account of this effect, we propose a novel approach that quantify the potential removal rate of MP in these areas by mimicking their behavior as those of lateral cavities (Jackson et al. 2013).

In particular, our newly proposed methodology accounts for the time a MP particle spends crossing a lateral cavity by defining a surface transient storage time ($\tau _{STS}$), assuming a rectangular shape for the lateral cavities, and by characterizing the number of the possible lateral cavities (i.e., the number of transient storage zones $n_{STS}$) a MP particle can experience traveling along a given river reach, thereby offering a new perspective on particle retention dynamics in riverine environments. This approach stems from the assumption that an increased number of lateral cavities within a river network, each adding to the storage time, significantly impacts the retention of contaminants. As the number of cavities and the time spent within those cavities increase, the rate of removal decreases proportionally. Within this framework, $k_{banks}$ can be characterized as follows:

15 $$\begin{aligned} k_{banks} = \frac{1}{n_{STS} \cdot \tau _{STS}} \end{aligned}$$

The interaction between water flow and lateral cavities along river reaches is crucial to determine the residence time of particles within the system, which in turn impacts the efficiency of MP removal. To characterize this interaction, we use the Reynolds number (*Re*), a key dimensionless parameter that indicates the flow regime within the river as either laminar or turbulent. It is calculated as follows:

16 $$\begin{aligned} Re = \frac{vY_{0}}{\nu } \end{aligned}$$

where $\nu \;[L^2T^{-1}]$ represents the kinematic viscosity.

Analyzing the dataset provided by Jackson (2010) and Jackson et al. (2013), which records the transient storage time ($\tau _{STS}$) and *Re* for various streams and rivers, we derive the following power law relationship:

17 $$\begin{aligned} \tau _{STS} = 0.0547 \cdot Re^{0.6722} \end{aligned}$$

**Fig. 3:**
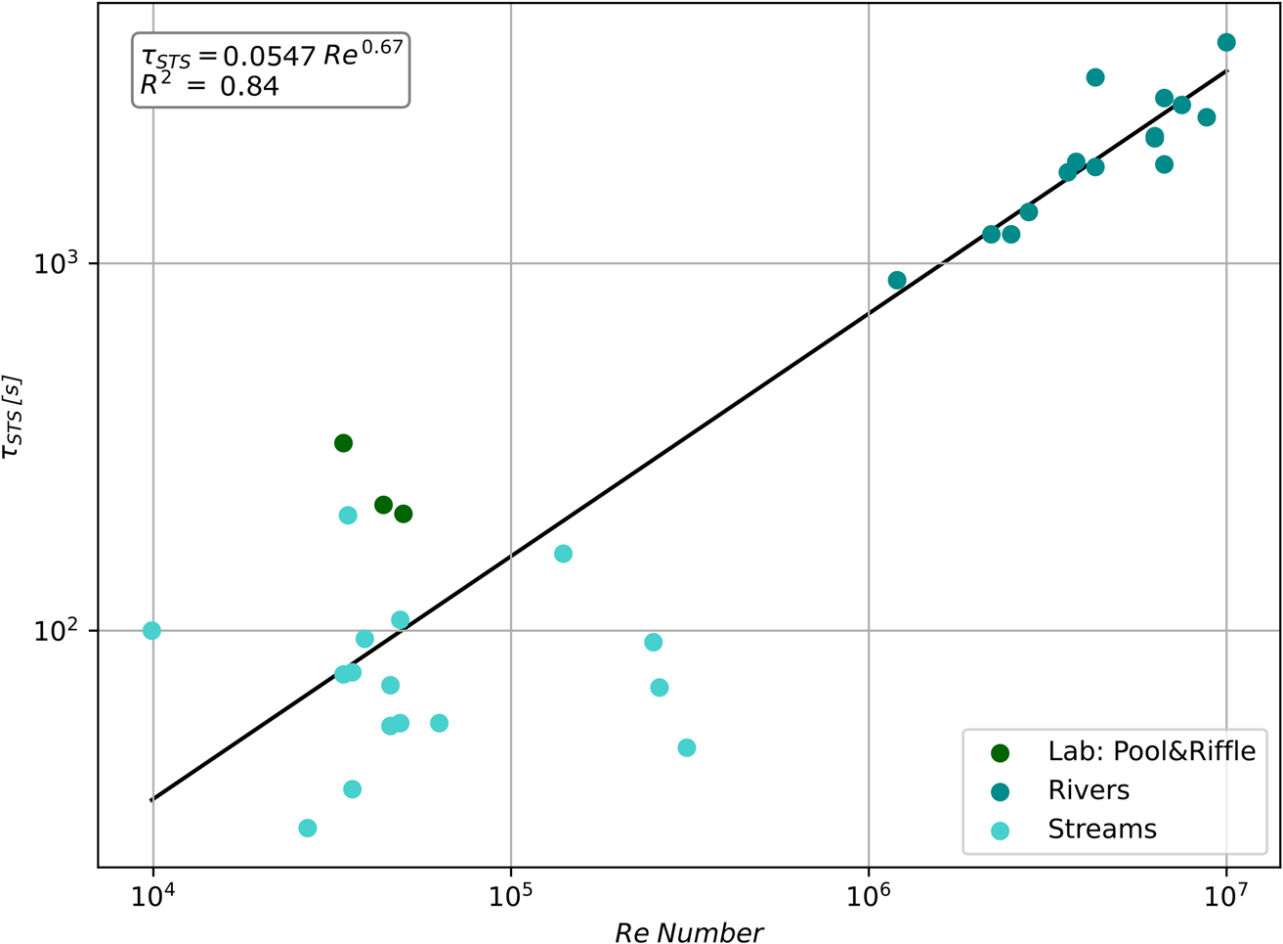
Relationship between the mean residence time of the transient storage zones and Reynolds’s number, based on the dataset from Jackson (2010)

The high value of the coefficient of determination ($R^2 = 0.84$) underlines the capability of the proposed power law model to represent the measured data, as shown in Fig. 3 and detailed in SI Table S1.

**Fig. 4:**
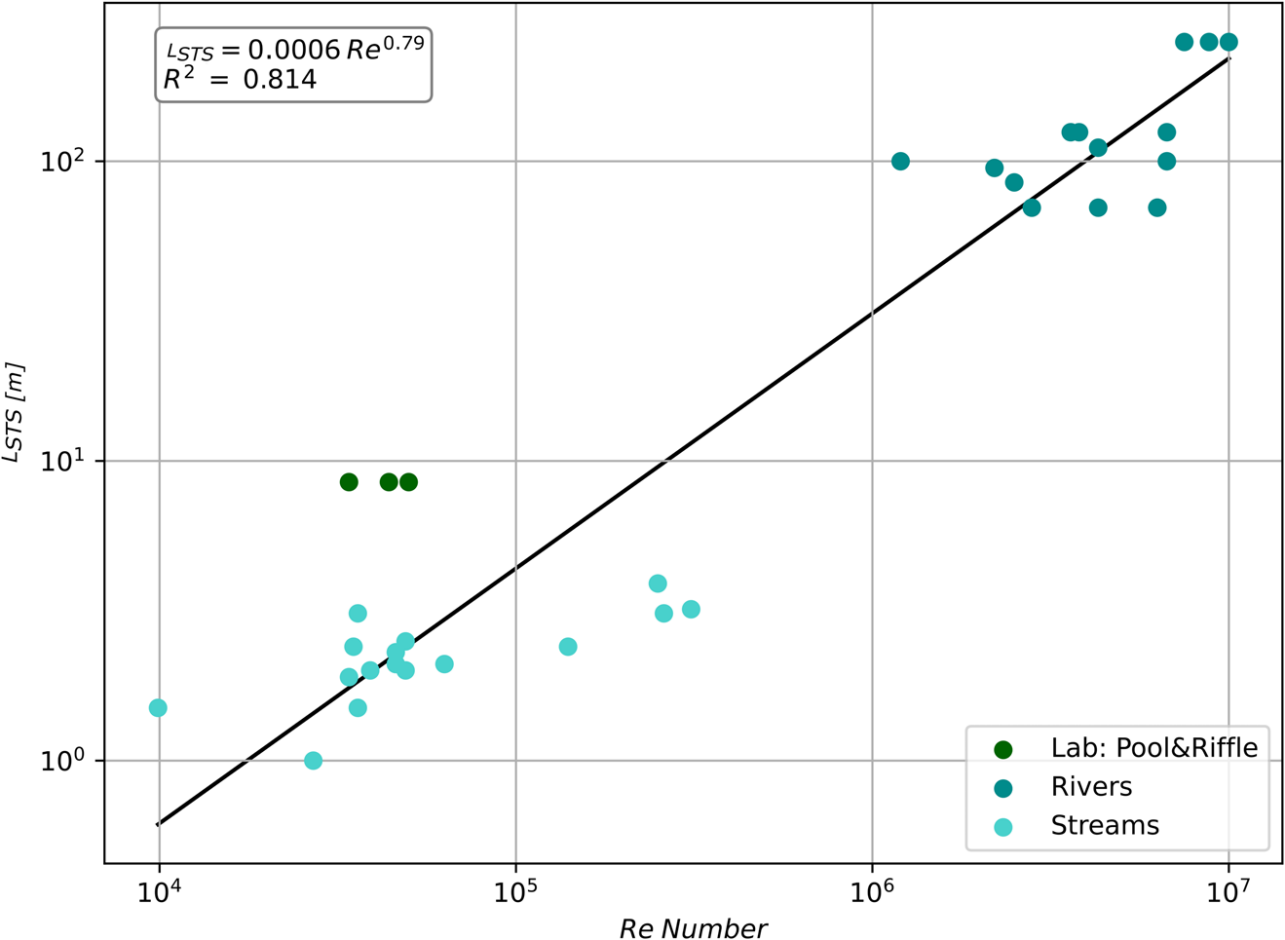
Relationship between the length of the transient storage zones and Reynolds’s number, based on the dataset from Jackson (2010)

The number of transient storage zones along a reach ($n_{STS}\;[-]$) is calculated using the following equation:

18 $$\begin{aligned} n_{STS} = \textrm{int}\left( \frac{x}{L_{STS}}\right) \end{aligned}$$

where $L_{STS}\;[L]$ is the length of a transient storage zone (refer to Fig. 4 and Table S1 estimated according to Eq. 18 ($R^2\,= \,0.81$), derived by using the same dataset (Jackson 2010; Jackson et al. 2013):

19 $$\begin{aligned} L_{STS} = 0.0006 \cdot Re^{0.79} \end{aligned}$$

The power law model proposed in Eq. 19 and showed in Fig. 4 is in accordance with other studies reporting that higher discharges lead to larger transient storage areas (Jung et al. 2024; Przyborowski et al. 2024).

It is important to emphasize that there is an inverse relationship between the rate of removal of MP and the removal time. The concept of inverse proportionality here means that as the opportunity of removal by sedimentation (time in the storage zone) increases, the fraction of easily settable particles decreases, thus reducing the rate at which particles are removed. It is a reflection of the changing composition of the particle population over time rather than a decrease in the physical capacity of the zone to remove MP particles.

#### Resuspension

The resuspension of MP particles from river networks refers to the frequency and extent to which MP are re-introduced into the water column from the riverbed. This process is influenced by various physical, chemical, and biological factors within the aquatic environment (i.e., water turbulence, bottom currents, and physical disturbances such as flood events) (Waldschläger and Schüttrumpf 2019; Francalanci et al. 2021). Our methodology integrates several methods, starting with the principle that a particle resuspension occurs only when the critical shear stress threshold is surpassed, with no resuspension happening below this point, the so-called Kronen-Partheniades method (Krone 1962; Partheniades 1965). This method was implemented by Quik et al. (2015) to model the fate of nanomaterials in river networks. Accordingly, the resuspension flux is defined as follows:where $R_j$ is the resuspension flux [$M L^{-2} T^{-1}$], $R_{j,max}$ is a constant specifying the maximum resuspension rate, $\tau$ represents the bed shear stress ($N m^{-2}$), and $\tau _{cr}$ is the critical shear stress required to initiate particle motion. $\tau$ is estimated as follows:

21 $$\begin{aligned} \tau = \rho _w \cdot g \cdot R_{h} \cdot S_f \end{aligned}$$

with $\rho _w$ the water density ($1000\;kg\;m^{-3}$), *g* the acceleration due to gravity ($9.81\;m\;s^{-2}$), $R_h$ the hydraulic radius [*L*], and $S_f$ the friction slope that, for the case of uniform open channel flows, equals the slope of the stream ($S\;[-]$).

The critical shear stress, $\tau _{cr}$, is given by Yu et al. (2023)’s formula modified from Waldschläger and Schüttrumpf (2019):

22 $$\begin{aligned} \tau _{cr} = \tau _{cs,50}\cdot 0.5588\left( \frac{\rho _{p}-\rho _w}{\rho _s - \rho _w}\right) \cdot \left( \frac{D_n}{D_{50}}\right) ^{0.497} \end{aligned}$$

Here, $\rho _{p}$ and $\rho _s$ are the densities of the plastic particle and sediment, respectively, while $D_n$ is the equivalent particle diameter and $D_{50}$ represents the median grain size of the sediment bed.

The resuspension rate ($k_{res}$) is then calculated using the theory of the nutrient spiraling system (see, e.g., Wollheim (2016) and citations therein) as follows:

23 $$\begin{aligned} k_{res} = \frac{R_j}{C_{MP}\cdot W} \end{aligned}$$

where $$C_{MP}$$ is the initial concentration of MP within the water column (i.e., $$C_{MP}=C_0$$) and *W* is the width of the channel. Finally, it is not accurate to assume an unlimited resuspension capacity of MP from the riverbed (Nizzetto et al. 2016b). To address this, constraints have been integrated into the model to reflect more realistic conditions. Resuspension is typically the dominant process in scenarios involving flooding or other extreme hydrological events (Gündoğdu et al. 2018). However, since our model is tailored for average flow conditions, the resuspension rate constant is deliberately constrained. Specifically, it cannot exceed the sum of the rate constants for burial, sedimentation, and bank removal. In scenarios where $$k = 0$$, this implies that the processes are in equilibrium, leading to a situation where only advection and dispersion processes are significant. This scenario is evaluated in detail in Portillo De Arbeloa and Marzadri (2024). It is important to note that, ideally, the resuspension flux should be directly influenced by the concentration of MP in the sediment rather than being solely determined by the MP concentration in the water column. However, due to the lack of comprehensive datasets quantifying MP concentrations in riverbed sediments across relevant spatial and temporal scales, our model does not explicitly include $$C_{MP,sed}$$. Instead, we assume that $$C_{MP} $$ is constant and equal to the initial MP concentration in each reach ($$C_{MP}$$ = $$C_0$$). In our framework, the fraction of MP that resides in the sediment and contributes to resuspension is indirectly incorporated within the $$R_j$$. This assumption ensures that deposition-resuspension dynamics are accounted for while working within current data limitations.

### Model implementation

The model delineated in “Mathematical model” was integrated into the framework established by Portillo De Arbeloa and Marzadri (2024). The initial model assumed equilibrium among transport processes within the water column (i.e., sedimentation, resuspension, and burial), with advective transport recognized as the primary mechanism driving MP mobility. Evaluated under mean average flow conditions, the model introduces a comprehensive system designed to quantify MP generation within the analyzed region. It estimates the initial MP concentration by assessing the volume of plastic waste produced by the local population, incorporating metrics such as the Human Development Index (HDI), per capita solid waste production, and the proportion of plastics in the solid waste generated (Mai et al. 2019). Recent empirical studies support the validity of this population-based estimation approach. Kunz et al. (2023) analyzing measured data on MP pollution along the main tributaries of the Wu river (Taiwan) found a positive correlation between MP abundance and population density, highlighting the influence of urbanization on MP distribution in freshwater systems. Similarly, Imbulana et al. (2024) observed that higher MP concentrations were associated with urban centers and industrial areas in Japan, suggesting that population density and related anthropogenic activities are significant predictors of MP pollution levels in river networks.

The algorithm employs open-source databases to map the dendritic structure of river networks (i.e., MERIT Yamazaki et al. 2019). Additionally, it estimates river characteristics, including width, mean flow depth, and average velocity, employing methodologies developed by Raymond et al. (2012) and the dispersion coefficient formulation by Saco and Kumar (2002). Discharge, on the other hand, can be inputted for every study reach or estimated through a power law relationship between the long-term discharge $Q; [L^3T^{-1}]$ and the catchment cumulative drainage area $(CDA;[L^2])$.

The algorithm initiates the process by simultaneously introducing the mass of MP being generated by each sub-basin across all starting nodes of the river reaches over a specified duration. We estimate the MP load on a daily basis and simulate it as a constant 24-h pulse, representing a uniform daily input reflective of continuous sources (i.e., wastewater treatment plants Murphy et al. 2016; Sun et al. 2019). This approach aligns with the common practice of reporting plastic consumption and waste generation on an annual basis, allowing for daily loads to be derived and providing a consistent temporal framework across sub-basins. The algorithm then applies the custom function to simulate the transport of these masses to subsequent downstream nodes. This methodology incorporates dilution effects by performing a mass balance at each confluence node (points where multiple nodes converge) to ensure precise modeling of MP transport (see Portillo De Arbeloa and Marzadri 2024 for more details). By including this mathematical approach that accounts for the transport processes of MP, we aim to provide a more realistic estimation of the dynamics of MP in river networks, building on existing geo-referenced contaminant mass balance frameworks such as that described by Keller et al. (2007), while adapting the structure to a node-based river network representation and processes specific to particulate transport.

The model outputs a breakthrough curve delineating the concentration of MP at a specified point of interest. The breakthrough curves reflect different peaks corresponding to the arrival of MP inputs from various upstream nodes, driven by their respective input concentrations and travel times. Given the scarcity of continuous MP concentration measurements within river networks, a direct comparison of our results with continuous MP concentration values in rivers is unfeasible (Blevins et al. 2021; Bruge et al. 2020). The discharge stage at the time of sample collection is also unknown. Hence, by comparing the average outcomes of the model’s breakthrough curve, we aim to bridge this information gap (Portillo De Arbeloa and Marzadri 2024). We performed scenario analyses to evaluate MP transport dynamics under varying sample composition conditions. We selected the River Tame watershed, previously investigated by Woodward et al. (2021), as a possible river network due to the availability of a detailed and publicly accessible dataset on MP composition in riverine environments, including information on particle types and shapes. This site allowed for the implementation of multiple realistic particle scenarios within a consistent framework. Furthermore, the River Tame drains a highly urbanized and industrialized catchment with multiple wastewater treatment plants along its corridor, making it broadly representative of European river systems with substantial anthropogenic MP inputs.

Additionally, the model incorporates the representation of the total injected MP mass into various MP types. Four distinct MP composition scenarios were implemented to assess how particle-type distribution affects transport outcomes: (1) original proportions from Woodward et al. (2021), representing typical site-specific conditions and high-fiber scenario; (2) a high-fragment scenario (Margenat et al. 2021; 3) a high-pellet scenario (Mani et al. 2015); and (4) a scenario derived from an averaged composition from recent literature (Table S2, Supplementary Information (Akdogan et al. 2023; Liu et al. 2023; Schrank et al. 2022; Yuan et al. 2022; Margenat et al. 2021; Woodward et al. 2021; Han et al. 2020; Mani et al. 2015)). All selected studies were conducted in European contexts, where similarities in socio-economic profiles and plastic consumption and waste treatment practices support the assumption of cultural and behavioral consistency in MP generation and management. The compositions applied in each scenario are summarized in Table 1.

**Table 1:** Proportions of microplastic types (fibers, pellets, fragments, and others) across four scenarios. Data are drawn from the studies by Woodward et al. (2021); Mani et al. (2015); Margenat et al. (2021), and from averaged values based on additional analyses

Type	Scenario 1	Scenario 2	Scenario 3	Scenario 4
Fibers	0.976	0.007	0.025	0.528
Pellets	0.012	0.040	0.584	0.096
Fragments	0.008	0.953	0.375	0.342
Others (i.e., films)	0.004	0.000	0.011	0.034
Total	1.000	1.000	0.995	0.999

Lastly, it is essential to define parameters like *csf* and *E* to establish a universal conversion factor between them for instances where not all dimensions of the particle are known. We calculated a relationship between *csf* and *E* from the SI dataset of Francalanci et al. (2021). The data points employed for this calculation are depicted in Fig. S1. The derived equation, is presented as follows (Eq. 24):

24 $$\begin{aligned} E = 1.1445 \cdot csf^{-0.097} \end{aligned}$$

**Table 2:** Average plastic density $(kg/m^3)$ according to their particle type

	$$\overline{\rho _p}$$
Fragment	1082.25
Fiber	1432.00
Pellets	2401.61
Others (i.e., films)	[981.50–1013]

Considered density of films was the maximum value to avoid all particles being buoyant

**Fig. 5:**
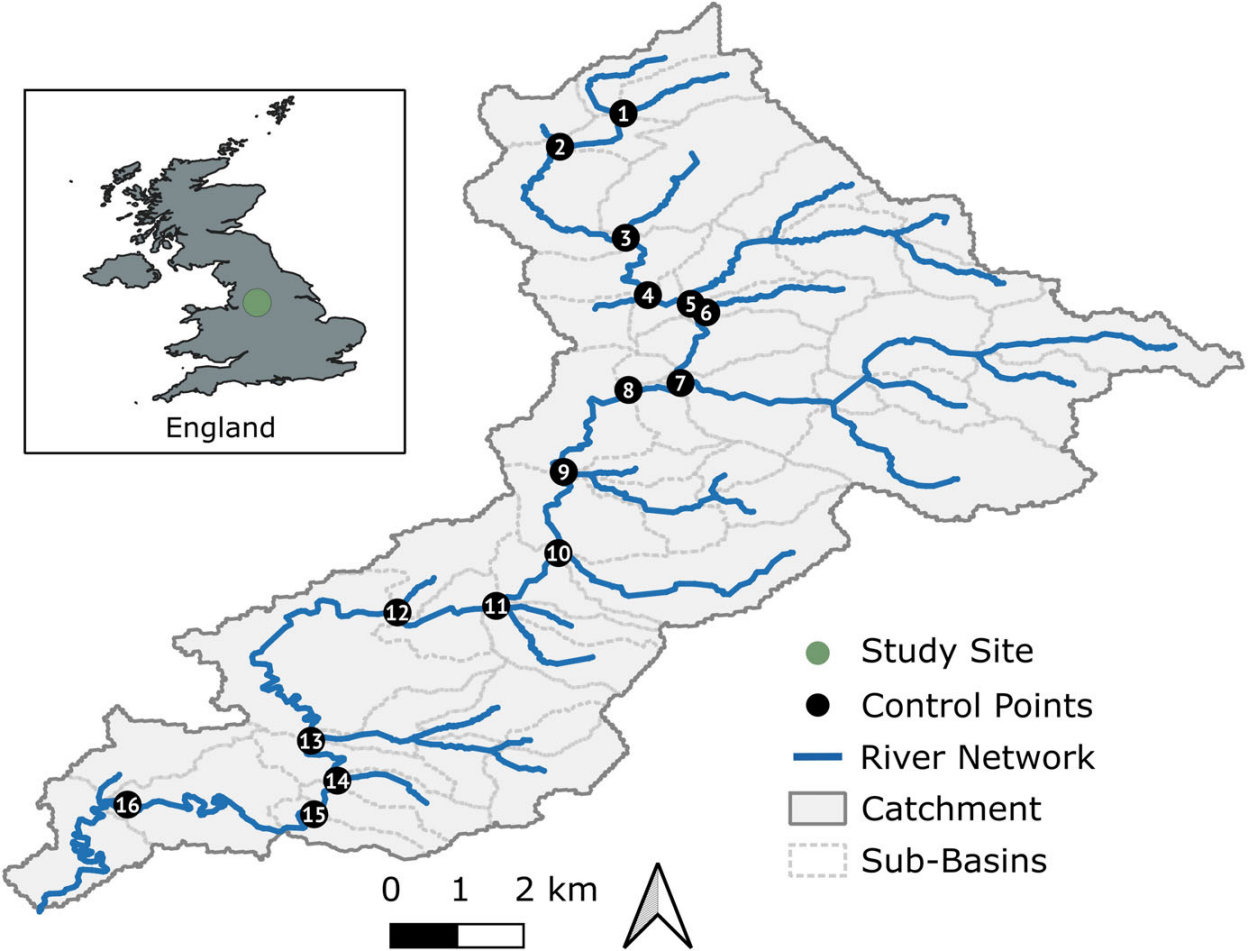
Map of the Tame River network (location of the study site in the left box) showing the river reaches, the relative sub-basins, and the identified control sections (control points) across the mainstem

To determine the appropriate *D* and *csf* values for each type of MP particle to be used in the model, we analyzed some existing MP particles datasets (Van Melkebeke et al. 2020; Le Roux 2004). The analysis focused on the four most commonly encountered types of MP particles (Kunz 2018): fibers, fragments, pellets, and films. To achieve this, we calculated key statistical indicators, specifically the 25th, 50th (median), and 75th percentiles, as illustrated in Figs. S2 and S3. These statistical analyses facilitated the derivation of varied *csf* and *D* value combinations, which were subsequently incorporated into our model to simulate diverse particle characteristics under different scenarios. The densities values used for the different types of MP are derived from the average values reported in the studies of Francalanci et al. (2021); Van Melkebeke et al. (2020), and Le Roux (2004) as listed in Table 2.

The final implementation of the model in the River Tame watershed considered all four scenarios and integrated type-specific density and morphological properties. The final breakthrough curve at each control section was derived by aggregating the results across different particle types, with a focus on the “Median-Median” combination. The control sections were defined as the nodes along the mainstem of the river network, allowing spatial analysis of how MP concentrations evolve as the pollutant moves downstream. The study by Woodward et al. (2021) serves as the foundation for analyzing our model outputs because it offers detailed information on MP types, composition, and concentrations, which are crucial for our assessment. The comprehensive data provided by this study enables us to examine the occurrence and distribution of MP in the environment with more precision. The Tame River (England) flows from rural headwaters to urbanized areas with a direct effect of land use on water quality. Urbanization impacts are evident, with five wastewater treatment works connected to over 221,000 people (Woodward et al. 2021). Figure 5 show the Tame River network, while the data used to estimate the MP load are presented in Table 3.

**Table 3:** Input data. *HDI* is the Human Development Index, *SWG* is the mass of solid waste generation per inhabitant, *PP* is the percentage of microplastic in the mass of solid waste

	HDI	SWG	PP
	$(-)$	(*Kg*/*inh*)	$(\%)$
Tame River	0.932	1.33	0.115

**Fig. 6:**
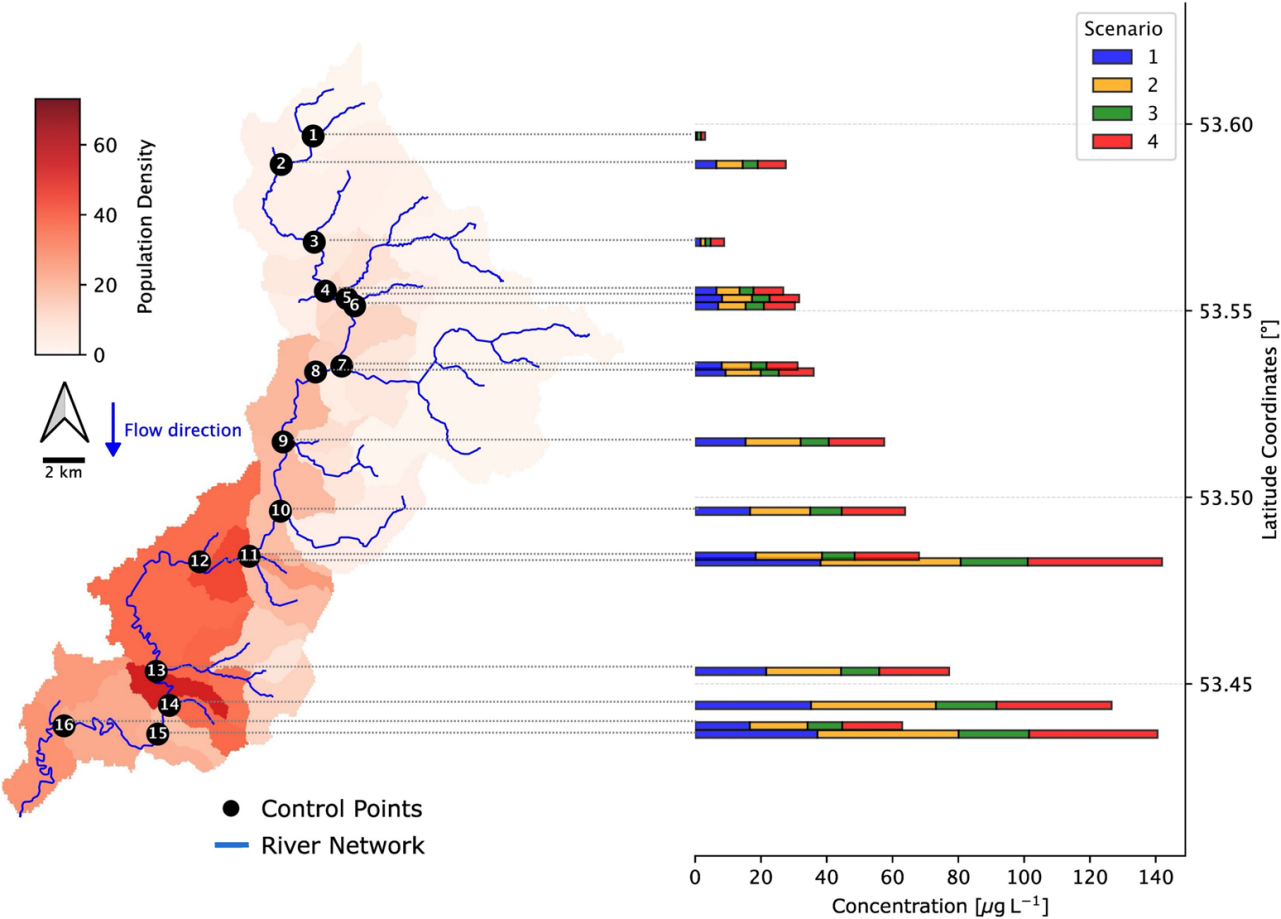
Spatial distribution of population density across the study catchment (left) and corresponding mean MP concentrations at successive control sections along the river mainstem (right) for the four modeled composition scenarios. Black circles mark the control sections on the map and are vertically aligned with their concentration values in the adjacent panel. The four scenarios are represented by colored-coded bars: Scenario 1 (blue), Scenario 2 (yellow), Scenario 3 (green), and Scenario 4 (red)

The river network and basin information used for the model were obtained from https://www.ordnancesurvey.co.uk/products/os-open-rivers. The measurement sites and basins can be seen in Fig. 5. Additionally, the input data to estimate the MP load is reported in Table 3. The *SWG* and the *P* were extracted from Kaza et al. (2018) while the *HDI* was extracted from Klugman et al. (2011). The population density was extracted from Schiavina et al. (2023).

## Results and discussion

The results focus on the application of the 1D ADRE model, which incorporates a first-order decay term to represent key retention and exchange processes including sedimentation, burial, resuspension, and bank retention. As described in “Methods,” the model employs watershed characteristics, demographic-based MP load estimates, hydromorphological features, and particle-specific attributes (i.e., shape, size, and density) to simulate MP transport in river networks. Among these processes, bank retention and sedimentation emerge as the dominant transport mechanisms, resulting in significant MP accumulation in low energy areas. In contrast, resuspension played a minor role in the modeled system, though we acknowledge its broader importance in MP transport. The model was implemented in the Tame River (England) (Woodward et al. 2021) using the input data for MP load estimation summarized in Table 3.

The modeled MP concentration profiles along the river mainstem exhibit clear spatial patterns (Fig. 6), influenced significantly by both particle-type composition and MP inputs driven by population density across sub-basins. In all evaluated scenarios, MP concentrations tend to progressively increase downstream, reflecting the cumulative addition of MP loads from upstream populated areas and the inherent transport characteristics of the particles. Scenarios 1 (high fibers, *blue bars* in Fig. 6), 2 (high fragments, *yellow bars*), and 4 (average composition with fiber predominance, *red bars*) all show relatively high MP concentrations downstream, though driven by different particle characteristics and input distributions. Each begins with moderate upstream concentrations (0.14–1.22 $\mu$g/L) and exhibits a steady increase toward downstream peaks, surpassing 35 $\mu$g/L for fibers, $\sim$42.9 $\mu$g/L for fragments, and $\sim$40.8 $\mu$g/L for the average composition. This downstream accumulation reflects both the particle-specific transport properties and significant MP contributions from densely populated basins. A spatially explicit map showing the modeled concentrations along the entire river network for Scenario 1 is included in the Supplementary Information (Fig. S4). This map may serve as a useful baseline for further pollutograph analysis using indicators such as the Pollution Load Index (PLI) (Ephsy and Raja 2023; Portillo De Arbeloa and Marzadri 2024; Wang et al. 2024) and could support the identification of MP hotspots across catchments.

Fibers (Scenario 1), characterized by their elongated shape and moderate density (1432 kg/m$^3$), remain suspended longer and travel farther, contributing to widespread persistence in the river network. Their shape enhances interaction with flow turbulence, increasing their possibility of resuspension and enabling long-range downstream mobility (Akdogan et al. 2023). Fragments (Scenario 2, *yellow bars* in Fig. 6), with a slightly lower density (1085.25 kg/m$^3$) and irregular shapes, exhibit even higher mobility, maintaining elevated concentrations along the entire mainstem. They also tend to be less easily resuspended once deposited, likely due to their compact geometry and lower surface area-to-volume ratios, which reduce their responsiveness to turbulent re-entrainment (Kowalski et al. 2016). The average composition scenario (Scenario 4, *red bars* in Fig. 6), dominated by fibers, follows a similar pattern of Scenario 2, though with slightly lower peaks, representing typical MP behavior in European rivers.

In contrast, Scenario 3 (high pellets, *green bars* in Fig. 6) exhibits significantly different dynamics. Concentrations begin at $\sim$1.27 $\mu$g/L but increase only modestly to $\sim$21.4 $\mu$g/L downstream. This limited downstream mobility is primarily attributed to the much higher density of pellets (2401.61 kg/m$^3$), which promotes rapid settling and deposition. Despite substantial MP inputs from upstream urban areas, pellets tend to accumulate locally, underscoring the critical role of density in determining MP fate and spatial distribution.

Across all scenarios, the increase in MP concentration along the mainstem is not uniformly gradual but characterized by abrupt peaks at certain control points. These often correspond to sub-basins with high population densities. Notably, drops in concentration near control points 4 and 14 coincide with the confluence of sub-basins that exhibit low population densities and high discharge, resulting in a dilution effect. In contrast, control point 13 displays a sharp peak, coinciding with a notable rise in local population density. Interestingly, order-1 sub-basins at points 14 and 15, despite their low populations, contribute to relatively high MP concentrations due to limited discharge, which reduces dilution capacity.

These findings highlight how MP concentrations are shaped by a combination of demographic and hydromorphological heterogeneity. Discharge plays a dual role—on one hand facilitating downstream transport and on the other acting as a dilution mechanism. Consequently, a highly populated sub-basin may not necessarily lead to high concentrations if its discharge is also high enough to dilute MP presence. Conversely, smaller tributaries with lower discharge but fewer inputs may exhibit higher localized concentrations. This interaction underscores the importance of accounting for both MP loading from population density and the flow capacity of receiving reaches when interpreting spatial patterns in MP concentrations.

Additional uncertainties may arise from factors not explicitly captured by the model. These include point-source discharges such as untreated wastewater, episodic stormwater runoff, or localized infrastructure influences like bridge crossings and urban drainage systems (Schoof 1980; Swadener et al. 2014; Bakr et al. 2020). Such sources can introduce MP independently of population density distributions, leading to elevated concentrations in specific reaches. Furthermore, continuous effluent discharges from wastewater treatment plants (WWTPs) are known contributors to MP loads and can dominate local conditions (Fendall and Sewell 2009; Schmidt et al. 2020). These complexities highlight the limitations of modeling frameworks that primarily rely on diffuse, demographic-based inputs, and underscore the need to complement such approaches with field observations and source specific assessments.

Taken together, these results reinforce the importance of modeling both particle properties and watershed-scale demographic and hydrological conditions. They also highlight the limitations of models that neglect localized or point-source pollution pathways. Effective MP management in riverine environments must therefore address both system-wide diffuse sources and highly localized inputs, while incorporating the interplay between transport dynamics, particle-specific retention, and dilution effects.

**Fig. 7:**
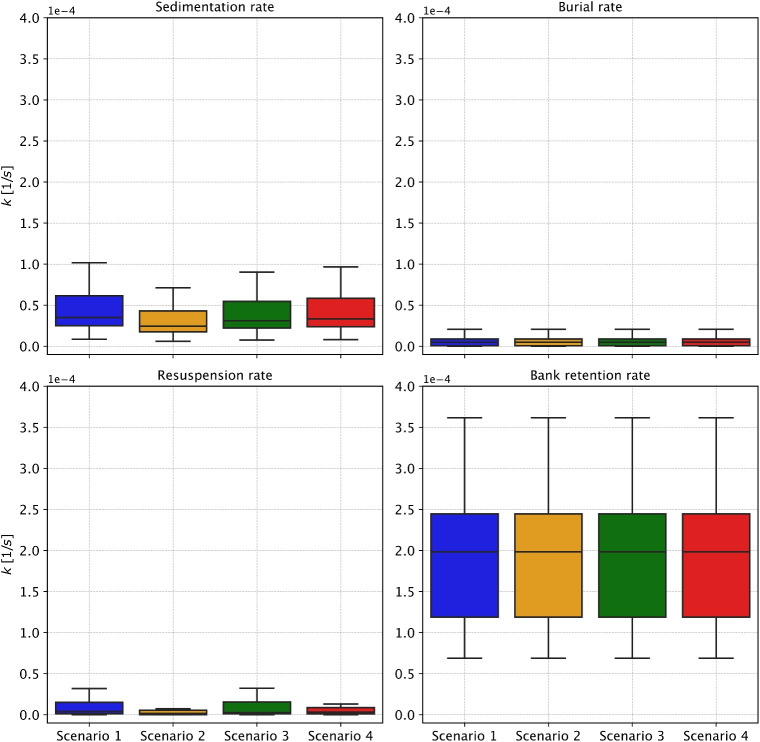
Distribution of transport process coefficient rates across the entire river network for all scenarios

While absolute MP concentration values vary between scenarios, the consistent dominance of sedimentation and bank retention processes indicates that MP predominantly accumulate in low-energy zones such as riverbanks and depositional areas. Figure 7 displays boxplots of modeled process rates across the river network under each scenario. Bank retention remains the most significant retention mechanism, showing limited variability across scenarios due to its independence from particle morphology. In contrast, sedimentation and resuspension rates exhibit noticeable variation between scenarios, as they are sensitive to particle characteristics such as density and shape. Scenario 3 (high pellets) shows slightly elevated sedimentation and reduced resuspension rates, reflecting the high density and low mobility of pellets. Scenario 1 (high fibers) exhibits the highest resuspension rates, consistent with the high mobility of fibers, while Scenario 2 (high fragments) yields the lowest sedimentation and resuspension, suggesting intermediate behavior. Scenario 4 (average composition) presents a balanced distribution across both processes. Burial consistently plays a minor role in MP retention across all cases.

The proposed approach for estimating bank retention rates is based on the premise that flow deceleration occurs along riverbanks, where shallower depths increase travel times and facilitate particle deposition (Pathak and Demars 2023). Studies have shown that contaminant accumulation is significantly higher near riverbanks than in central channels, reinforcing their role as key retention zones for suspended particles (Baborowski et al. 2004). This localized retention can influence both short-term MP transport and long-term accumulation, as MP may either settle permanently or be remobilized depending on hydrological conditions. Based on the findings of Gücker and Boëchat (2004), surface transient storage, a component frequently overlooked in stream tracer research, plays a pivotal role in pollutant retention. The authors emphasize that channel morphology and transient storage zones, such as eddies and bank indentations, significantly influence the transport and fate of contaminants by trapping particles in low-energy environments. A similar retention mechanism has been observed in groyne fields, where low-velocity zones encourage the deposition of fine sediments and associated pollutants, including MP (Baborowski et al. 2004). These structures, much like natural riverbanks, act as temporary sinks where pollutants can accumulate during low and moderate flow conditions, only to be remobilized during high-flow events. This evidence highlights the importance of hydromorphological influences on MP fate, with riverbanks serving as dynamic interfaces where deposition and resuspension processes interact where the retention capacity of these zones varies on factors such as sediment grain size, and local hydrodynamic conditions.

Transformation processes such as agglomeration, hetero-aggregation, homoaggregation, degradation, and biofouling (Kooi et al. 2018) play a crucial role in the fate of MP in river networks. Although not explicitly modeled, these processes influence MP behavior by altering size, buoyancy, and reactivity (Kooi et al. 2018). Biofouling, for example, increases MP density, promoting sedimentation, whereas defouling and degradation can enhance resuspension (Kooi et al. 2017; Leiser et al. 2020; Parrella et al. 2024). Additionally, uncertainties arise from the use of power law relationships to estimate streamflow and hydraulic parameters such as river width and depth (Portillo De Arbeloa and Marzadri 2024). These relationships rely on averaged data, which may not fully capture seasonal variability and extreme hydrological events. Moreover, the 1D ADRE assumes a homogeneous lateral and vertical MP distribution, potentially oversimplifying transport dynamics (Owowenu et al. 2023). However, given the lack of high resolution MP concentration data, this assumption is necessary to enable a mass balance approach across connected river reaches. Despite these limitations, the model effectively simulates MP transport by integrating hydromorphological attributes and MP particle characteristics.

Further improvements could focus on refining the formulation of resuspension and burial rates, as their current representation appears to have a limited impact on overall modeling outcomes. Specifically, the model constrains resuspension to MP concentrations above a threshold, leading to disproportionately high rates at very low concentrations an unrealistic scenario. Future research should investigate local scale interactions that govern resuspension and burial, considering the interplay of environmental conditions, particle properties, and hydrodynamics. A more detailed understanding of these processes would improve model accuracy and contribute to more effective risk assessment and management strategies for MP pollution in fluvial systems.

## Conclusions

River networks serve as conduits between terrestrial sources and marine environments, critically shaping the fate and distribution of MP. The modeling approach presented here is transferable and can be applied to other river networks, provided that relevant hydromorphological, demographic, and MP characterization data are available. This study advances previous modeling efforts by incorporating compositional scenarios that account for imbalances in transport processes and particle-specific properties. Using a 1D advection-dispersion-reaction equation (ADRE) model, we integrated key environmental mechanisms: (i) sedimentation, (ii) burial, (iii) resuspension, and a novel bank retention formulation to simulate MP fate across the Tame River network. Among these, bank retention emerged as the dominant retention process, demonstrating substantial influence on downstream MP accumulation in low-energy lateral zones.

The scenario-based framework allowed for an explicit comparison of MP dynamics across a range of particle morphologies and densities, reflecting realistic combinations observed in freshwater environments. Fibers and fragments exhibited high mobility and persistence, while denser particles such as pellets settled more rapidly, underscoring the role of physical properties in transport outcomes. Coupled with population-based MP loading and discharge estimates, the model revealed that spatial MP concentrations result from the interplay of source magnitude and local hydrodynamics. Specifically, high population density does not always yield high MP concentrations—tributary discharge regulates local dilution and transport efficiency, creating zones of both accumulation and dispersal.

While the model simplifies MP dynamics by assuming longitudinal homogeneity and excluding transformation processes, it nevertheless provides a robust basis for understanding spatial MP patterns. These simplifications are justified given the lack of continuous monitoring data and the need to achieve tractable mass balance estimates across large-scale river systems. Still, further improvements are needed. Future work should refine the resuspension and burial formulations, incorporate particle transformations such as aggregation, degradation, and biofouling, and explore interactions at the local scale. This would increase the predictive power of the model and enable more targeted mitigation strategies.

The findings of this study have practical relevance for environmental management and policy. For instance, scenario-informed pollution load indices could be used to develop spatial MP risk maps, guiding monitoring efforts and remediation priorities. By combining reach-scale hydromorphology, particle-specific attributes, and mechanistic transport processes, this study provides a valuable framework for assessing MP dynamics in riverine systems and supports evidence-based decision-making to mitigate MP pollution from source to sea.

## Glossary

*a*: Length of the longest particle axis (*L*)
*b*: Length of the intermediate particle axis (*L*)
$$\beta $$
Aspect ratio (−)
*C*: Concentration in the reach ($ML^{-3}$)
$$C_0$$
Initial concentration ($$ML^{-3}$$)
$$C_1$$
Constant dependent on the shape of the particle (−)
$$C_2$$
Constant dependent on the shape of the particle (−)
$$C_z$$
Dimensionless Chezy coefficient (−)
*c*: Length of the shortest particle axis (*L*)
*csf*: Corey Shape Factor (−)
*D*: Particle diameter (*L*)
$$D_L$$
Hydrodynamic dispersion coefficient ($L^2T^{-1}$)
$$D_{50}$$
Mean grain size of sediments (*L*)
$$d_s$$
Relative submergence (−)
*E*: Grain shape factor (−)
*g*: Gravitational acceleration ($$LT^{-2}$$)
*H*(*t*): Heaviside step function (−)
$$H_d$$
Dune height (*L*)
$$h_m$$
Amplitude of water depth oscillation (*L*)
*k*: First-order decay rate ($T^{-1}$)
$$k_{banks}$$
Banks retention rate ($T^{-1}$)
$$k_{bur}$$
Burial rate ($$T^{-1}$$)
$$k_{res}$$
Resuspension rate ($$T^{-1}$$)
$$k_{sed}$$
Sedimentation rate ($$T^{-1}$$)
$$K_h$$
Hydraulic conductivity of the streambed sediment ($LT^{-1}$)
$$L_{STS}$$
Estimated length of a storage zone (*L*)
$$n_{STS}$$
Number of transient storage zones along a reach (−)
$$\nu $$
Kinematic viscosity of water ($L^2T^{-1}$)
$$\overline{q}_{HZ}$$
Mean hyporheic downwelling flux ($LT^{-1}$)
$$\phi $$
Streambed porosity (−)
*R*: Relative density (−)
*Re*: Reynolds number (−)
$$\rho _p$$
Density of the plastic particle ($ML^{-3}$)
$$\rho _w$$
Density of water ($ML^{-3}$)
*S*: Stream slope (*L*)
*t*: Time (*T*)
$$t_c$$
Time span of MP release (*T*)
$$\tau _{STS}$$
Transient storage time of an MP particle within lateral cavities (*T*)
*v*: Mean flow velocity ($LT^{-1}$)
$$v_s$$
Settling velocity ($$LT^{-1}$$)
$$v_w$$
Kinematic wave celerity ($LT^{-1}$)
*W*: Channel’s width (*L*)
*x*: Length of the reach (*L*)
$$Y_0$$
Mean flow depth (*L*)
$$Y_{hz}$$
Hyporheic zone depth (*L*)
$$Y_s$$
Dimensionless bedform height (−)

## Supplementary information

Contains some of the relevant datasets used in the research and analysis presented herein.

## Supplementary Information

Below is the link to the electronic supplementary material.

Supplementary file 1 (pdf 923 KB)

## Data Availability

The authors declare that the data supporting the findings of this study are available within the paper and in its supplementary information files.
