# Supplementary material for: Modeling microplastic dynamics in riverine systems: fate and transport analysis
Source: Environ Sci Pollut Res Int. 2025 Aug 22;32(34):20659–77. doi: 10.1007/s11356-025-36875-6 (PMC12431913; doi:10.1007/s11356-025-36875-6)
Supplement: Supplementary file 1 — (pdf 923 KB) [file 11356_2025_36875_MOESM1_ESM.pdf]

Modeling Microplastic Dynamics in Riverine Systems: Fate and Transport Analysis

Nerea Portillo De Arbeloa[1], Alessandra Marzadri[1]

[1]University of Trento Department of Civil Environmental and Mechanical Engineering: Universita degli Studi di Trento

Dipartimento di Ingegneria Civile Ambientale e Meccanica ITALY, Trento, Italy

Contents of this file

1. Burial
2. Sedimentation
3. Bank Retention
4. Discharge Data
5. Literature-Based MP Composition
6. Advection Dispersion Reaction Model
7. Figures S1 to S3
8. Table S1
9. Table S2

1. Burial

To assess the streambed morphological classification of a river reach, we utilize the median grain size of streambed material (d_{50}) and the gradient of the stream (S), following

---

Corresponding authors: N. Portillo De Arbeloa, (nk.portillodearbeloa@unitn.it); A. Marzadri, (alessandra.marzadri@unitn.it)

the categorization outlined by Montgomery and Buffington (1997). The criteria for each morphological type are specified as follows:

- For dune morphology: $S \leq 0.009$ and $d_{50} < 4$ mm.
- For pool-riffle morphology: $0.009 < S \leq 0.05$ and $d_{50} > 4$ mm.
- For step-pool and cascade morphology: $S > 0.05$ and $d_{50} > 4$ mm.

The estimation of (d_{50}) is based on the formulation provided by Lee and Julien (2006), as shown in Equation S1, which incorporates the bankfull discharge (Q), the stream gradient (S), and the critical Shield's parameter (θ_c). For streambeds possessing an armor ratio, defined as the ratio of the median grain sizes of the surface to subsurface materials, of 1.8, θ_c is set to 0.05, consistent with experimental observations in natural river settings (Andrews, 1983). The applicability of Equation S1 has been corroborated by Marzadri et al. (2021), who confirmed its strong agreement with values documented in the literature, thereby justifying its selection for our analysis.

$$d_{50} = \left(\frac{S}{4.981 \cdot Q^{-0.346} \cdot \theta_c^{0.966}} \right)^{1.047} \tag{S1}$$

Furthermore, the hydraulic conductivity (K_H) was determined using Equation 8 from the study by Salarashayeri and Siosemarde (2012), which depends on the granulometry of the streambed. The efficacy of this equation was also tested by Marzadri et al. (2021) through a comparison with a suite of empirical formulations and the ranges of values reported in the literature by Bouwer (1978).

1.1. Dune Morphology

Using the empirical formula to calculate the half amplitude of dynamic head variation proposed in Elliott and Brooks (1997) and by assuming that the dune height is dependent

of the mean flow depth according to the expression from Yalin (1964) ($H_d = 0.167Y_0$) we have:

$$h_m = \frac{0.28V^2}{2g}\left(\frac{H_d}{Y_0}\right)^{3/8} \tag{S2}$$

$$L_{bed} = 6 \cdot Y_0 \tag{S3}$$

1.2. Pool-Riffle Morphology

$$Y_s = \frac{1}{0.18d_s^{0.45}\beta^{1.45}} \tag{S4}$$

$$d_s = \frac{d_{50}}{Y_0} \tag{S5}$$

$$\beta = \frac{2W}{(Y_0)} \tag{S6}$$

$$L_{bed} = 6.5 \cdot W \tag{S7}$$

2. Sedimentation

Fig. S1 was used to estimate a relationship between the grain shape factor (E) and the Corey shape factor. Moreover a boxplot of the *csf* and equivalent diameteres used for the different types of MP particles can be seen in Figures S2 and S3

3. Bank Retention

Table S1 presents the dataset used to estimate the relationship between transient storage time, characteristic length, and Reynolds number, as reported by Jackson (2010)

4. Discharge Estimation

Based on Table 2 from Burgers, Schipper, and Jan Hendriks (2014), we have extracted the power-law formulation to describe the relationship between the cumulative drainage area (CDA [km^2]) and the discharge (Q [m^3/s]) as follows:

$$Q = 0.09838 CDA^{0.78} \tag{S8}$$

5. Literature Based MP Composition

To provide additional context, we compiled average MP type proportions from existing literature (Akdogan et al., 2023; Liu et al., 2023; Yuan et al., 2022; Han et al., 2020; Mani et al., 2015; Woodward et al., 2021; Margenat et al., 2022; Schrank et al., 2022; Kunz et al., 2023). These values may be useful for comparative studies or cases where empirical MP proportions are unavailable. The densities values used for the different types of MP are derived from the average values reported in the studies of Van Melkebeke, Janssen, and De Meester (2020) and Le Roux (2004), as showed in Figures S1-S3.

6. ADRE Model

The Advection Dispersion Reaction Equation (ADRE), together with the chosen boundary and initial conditions (Eq.s 5), can be formulated in the Laplace Space as follows:

$$\begin{aligned} & s\,\hat{C} + v\frac{d\hat{C}}{dx} - D_L\,\frac{d^2\hat{C}}{dx^2} + k\hat{C} = 0 \\ & \hat{C}(0,s) = \frac{C_0}{s}\left(1 - \exp\left(-\frac{t_c}{s}\right)\right) \\ & \left.\frac{d\,\hat{C}}{dx}\right|_{x\to\infty} = 0 \end{aligned} \tag{S9}$$

where $\hat{C}$ is the MP concentration in the Laplace space and s is the transformation variable.

The general solution to the transformed ADRE equation (Eq. S9a) is as follows:

$$\hat{C}(x,s) = c_1 \exp\left(\frac{vx}{2D_L}\left(-1 - \sqrt{1 - \frac{4D_L(k+s)}{v^2}}\right)\right) + c_2 \exp\left(\frac{vx}{2D_L}\left(1 + \sqrt{1 - \frac{4D_L(k+s)}{v^2}}\right)\right) \tag{S10}$$

to evaluate the integration constants, c_1 and c_2 must satisfy the boundary conditions in Eq.s S9b and S9c. Deriving with respect to x yields:

$$\Rightarrow \frac{C_1}{2}\left(\frac{v}{D_L} - \frac{1}{\sqrt{D_L}}\sqrt{\frac{v^2}{4D_L} + k + s}\right)\exp\left(\frac{vx}{2D_L}\right)\exp\left(\frac{x}{D_L}\sqrt{\frac{v^2}{4D_L} + k + s}\right) + \frac{C_2}{2}\left(\frac{v}{D_L} + \frac{1}{\sqrt{D_L}}\sqrt{\frac{v^2}{4D_L} + k + s}\right)\exp\left(\frac{vx}{2D_L}\right)\exp\left(\frac{x}{D_L}\sqrt{\frac{v^2}{4D_L} + k + s}\right) = 0 \tag{S11}$$

When $x \to \infty$, the first term tends to zero. While in the second term, to meet the condition in Eq. S9b, c_2 must be equal to 0.

$$\Rightarrow \hat{C}(x,s) = C_1 \exp\left(\frac{vx}{2D_L}\right)\exp\left(-\frac{x}{\sqrt{D_L}}\sqrt{\frac{v^2}{4D_L} + k + s}\right) \tag{S12}$$

Replacing S9b

$$\Rightarrow \hat{C}(0,s) = \frac{C_0}{s}\left(1 - \exp\left(-\frac{t_c}{s}\right)\right) \tag{S13}$$

$$C_1 = \frac{C_0}{s}\left(1 - \exp\left(-\frac{t_c}{s}\right)\right) \tag{S14}$$

In the Laplace space the solution of the problem in Eq.S9 is:

$$\hat{C}(x,s) = \frac{C_0}{s}\left[1 - \exp(-\frac{t_c}{s})\right]\exp\left(\frac{vx}{2D_L}\right)\exp\left(-\frac{x}{D_L}\sqrt{\frac{v^2}{4D_L} + k + s}\right) \tag{S15}$$

Or equivalently:

$$\hat{C}(x,s) = \frac{C_0}{s}\exp\left(\frac{vx}{2D_L}\right)\exp\left(-\frac{x}{D_L}\sqrt{\frac{v^2}{4D_L}+k+s}\right) - \frac{C_0}{s}\exp\left(-\frac{t_c}{s}\right)\exp\left(\frac{vx}{2D_L}\right)\exp\left(-\frac{x}{D_L}\sqrt{\frac{v^2}{4D_L}+k+s}\right) \quad \text{(S16)}$$

The solution in the real space can be obtained by the inverse Laplace transform of the solution in Eq. S15. Following Van Genuchten (1981), the solutions of a first order type boundary conditions, particularly case B1 with $C_i = 0$, $R = 1$, $\lambda = 0$, $\mu = k$, we have:

$$\begin{aligned} C(x,t) &= C_0\widetilde{H}(x,t) && \text{if } 0 < t \leq t_c \\ C(x,t) &= C_0\widetilde{H}(x,t) - C_0\widetilde{H}(x,t-t_c) && \text{if } t > t_c \end{aligned} \quad \text{(S17)}$$

$\hat{H}(x,t)$ is defined by Van Genuchten (1981) (Refer to Eq. 27 in (Van Genuchten, 1981)), therefore:

$$\begin{aligned} g(x,t) =& \frac{1}{2}\exp\left(-\frac{vx}{2D_L}\right)\exp\left(-\frac{vx}{2D_L}\sqrt{1+\frac{4kD_L}{v^2}}\right)\operatorname{erfc}\left(\frac{x - vt\sqrt{1+\frac{4kD_L}{v^2}}}{2\sqrt{D_L t}}\right) \\ &+ \frac{1}{2}\exp\left(\frac{vx}{2D_L}\right)\exp\left(-\frac{vx}{2D_L}\sqrt{1+\frac{4kD_L}{v^2}}\right)\operatorname{erfc}\left(\frac{x + vt\sqrt{1+\frac{4kD_L}{v^2}}}{2\sqrt{D_L t}}\right) \\ &\text{if } 0 < t \leq t_c \end{aligned} \quad \text{(S18a)}$$

$$\begin{aligned} g(x,t) =& \frac{1}{2}\exp\left(-\frac{vx}{2D_L}\left(1-\sqrt{1+\frac{4kD_L}{v^2}}\right)\right)\operatorname{erfc}\left(\frac{x - vt\sqrt{1+\frac{4kD_L}{v^2}}}{2\sqrt{D_L t}}\right) \\ &+ \frac{1}{2}\exp\left(-\frac{vx}{2D_L}\left(1+\sqrt{1+\frac{4kD_L}{v^2}}\right)\right)\operatorname{erfc}\left(\frac{x + vt\sqrt{1+\frac{4kD_L}{v^2}}}{2\sqrt{D_L t}}\right) \\ &- \frac{1}{2}\exp\left(-\frac{vx}{2D_L}\left(1-\sqrt{1+\frac{4kD_L}{v^2}}\right)\right)\operatorname{erfc}\left(\frac{x - (t-t_c)v + \sqrt{1+\frac{4kD_L}{v^2}}}{2\sqrt{D_L(t-t_c)}}\right) \\ &- \frac{1}{2}\exp\left(-\frac{vx}{2D_L}\left(1+\sqrt{1+\frac{4kD_L}{v^2}}\right)\right)\operatorname{erfc}\left(\frac{x + (t-t_c)v + \sqrt{1+\frac{4kD_L}{v^2}}}{2\sqrt{D_L(t-t_c)}}\right) \\ &\text{if } t > t_c \end{aligned} \quad \text{(S18b)}$$

The same solution has been reported in Fan, Fleischmann, Collischonn, Ames, and Rigo (2015). Additionally, for the cases where **Pe** $\longrightarrow \infty$, advective-dominated problem, the analytical solution for the step pulse injection after applying the Laplace transform is:

$$C(x,t) = C_0 \exp\left(-\frac{kx}{v}\right)\left[H\left(t-\frac{x}{v}\right) - H\left(t-\frac{x}{v}-t_c\right)\right] \tag{S19}$$

Likewise, in scenarios in which an **instantaneous injection** is assumed, the set of equations 5 is transformed into the Laplace space as:

$$\hat{C}(0,s) = C_0 \tag{S20a}$$

$$\left.\frac{d\hat{C}}{dx}\right|_{x\to\infty} = 0 \tag{S20b}$$

$$\hat{C}(x,s) = C_1 \exp\left[\frac{vx}{2D_L} - \frac{x}{\sqrt{D_L}}\sqrt{k+s+\frac{v^2}{4D_L^2}}\right] + C_2 \exp\left[\frac{vx}{2D_L} + \frac{x}{\sqrt{D_L}}\sqrt{k+s+\frac{v^2}{4D_L^2}}\right] \tag{S21}$$

$$\left.\frac{\partial\hat{C}}{\partial x}\right|_{x\to\infty} \Rightarrow C_2 = 0 \tag{S22}$$

$$\hat{C}(0,s) = C_0 \Rightarrow C_1 = C_0 \tag{S23}$$

$$\Rightarrow \hat{C}(x,s) = C_0 \exp\left[\frac{vx}{2D_L}\right] \exp\left[-\frac{x}{\sqrt{D_L}}\sqrt{k+\frac{v^2}{4D_L^2}}\right] \tag{S24}$$

Returning to the real domain, as outlined by Chandran (2001), the equation becomes:

$$\mathcal{L}^{-1}\left\{\exp\left(-k\sqrt{s+\alpha}\right)\right\} = \frac{k}{2\sqrt{\pi}t^{3/2}} \cdot \exp\left(-\alpha t - \frac{k^2}{4t}\right)$$

With $k = x/\sqrt{D_l}$ and $\alpha = k + v^2/4D_L$ the analytical solution becomes:

$$C(x,t) = \frac{C_0 x}{2\sqrt{\pi D_L} t^{3/2}} \exp\left[-\frac{(vt-x)^2}{4D_L t}\right] \exp\left[-kt\right] \tag{S25}$$

When neglecting dispersion (high **Pe**) the analytical solution for an **instantaneous injection** is as follows:

$$C(x,t) = C_0 \exp\left(-\frac{kx}{v}\right) H\left(t - \frac{x}{v}\right) \tag{S26}$$

References

Akdogan, Z., Guven, B., & Kideys, A. E. (2023). Microplastic distribution in the surface water and sediment of the Ergene River. *Environmental Research*, *234*, 116500. doi: 10.1016/j.envres.2023.116500

Andrews, E. D. (1983). Entrainment of gravel from naturally sorted riverbed material. *Geological Society of America Bulletin*, *94*(10), 1225. doi: 10.1130/0016-7606(1983)94⟨1225:EOGFNS⟩2.0.CO;2

Bouwer, H. (1978). *Groundwater hydrology.* New York [usw.], Düsseldorf: McGraw-Hill.

Burgers, H. E. R., Schipper, A. M., & Jan Hendriks, A. (2014). Size relationships of water discharge in rivers: Scaling of discharge with catchment area, main-stem length and precipitation. *Hydrological Processes*, *28*(23), 5769–5775. doi: 10.1002/hyp.10087

Chandran, P. (2001). Inverse Laplace transforms of a class of non-rational fractional functions. *International Journal of Mathematical Education in Science and Technology*, *32*(1), 136–140. doi: 10.1080/00207390121063

Elliott, A. H., & Brooks, N. H. (1997). Transfer of nonsorbing solutes to a streambed with bed forms: Theory. *Water Resources Research*, *33*(1), 123–136. doi: 10.1029/96WR02784

Fan, F. M., Fleischmann, A. S., Collischonn, W., Ames, D. P., & Rigo, D. (2015). Large-scale analytical water quality model coupled with gis for simulation of point

sourced pollutant discharges. *Environmental Modelling and Software*, *64*, 58-71. doi: 10.1016/j.envsoft.2014.11.012

Han, M., Niu, X., Tang, M., Zhang, B.-T., Wang, G., Yue, W., ... Zhu, J. (2020). Distribution of microplastics in surface water of the lower Yellow River near estuary. *Science of The Total Environment*, *707*, 135601. doi: 10.1016/j.scitotenv.2019.135601

Jackson, T. R. (2010). *Evaluation of the Hydraulic Connection between streams and aquifers at Baker and Snake Creek near Great Basin National Park, Snake Valley, White Pine County, Nevada* (Thesis).

Kunz, A., Schneider, F., Anthony, N., & Lin, H.-T. (2023). Microplastics in rivers along an urban-rural gradient in an urban agglomeration: Correlation with land use, potential sources and pathways. , *321*, 121096. Retrieved from `https://linkinghub.elsevier.com/retrieve/pii/S0269749123000982` doi: 10.1016/j.envpol.2023.121096

Lee, J.-S., & Julien, P. Y. (2006). Downstream Hydraulic Geometry of Alluvial Channels. *Journal of Hydraulic Engineering*, *132*(12), 1347–1352. doi: 10.1061/(ASCE)0733-9429(2006)132:12(1347)

Le Roux, J. (2004). A Hydrodynamic Classification of Grain Shapes. *Journal of Sedimentary Research*, *74*(1), 135–143. doi: 10.1306/060603740135

Liu, Q., Xiong, X., Wang, K., Wang, H., Ling, Y., Li, Q., ... Wu, C. (2023). Homogenization of microplastics in alpine rivers: Analysis of microplastic abundance and characteristics in rivers of Qilian Mountain, China. *Journal of Environmental Management*, *340*, 118011. doi: 10.1016/j.jenvman.2023.118011

Mani, T., Hauk, A., Walter, U., & Burkhardt-Holm, P. (2015). Microplastics profile along the Rhine River. *Scientific Reports*, *5*(1), 17988. doi: 10.1038/srep17988

Margenat, H., Nel, H. A., Stonedahl, S. H., Krause, S., Sabater, F., & Drummond, J. D.

(2022). Hydrologic controls on the accumulation of different sized microplastics in the streambed sediments downstream of a wastewater treatment plant (Catalonia, Spain). , *16*(11), 115012. Retrieved from https://iopscience.iop.org/article/10.1088/1748-9326/ac3179 doi: 10.1088/1748-9326/ac3179

Marzadri, A., Amatulli, G., Tonina, D., Bellin, A., Shen, L. Q., Allen, G. H., & Raymond, P. A. (2021). Global riverine nitrous oxide emissions: The role of small streams and large rivers. *Science of The Total Environment*, *776*, 145148. doi: 10.1016/j.scitotenv.2021.145148

Montgomery, D. R., & Buffington, J. M. (1997). Channel-reach morphology in mountain drainage basins. *Geological Society of America Bulletin*, *109*(5), 596–611. doi: 10.1130/0016-7606(1997)109⟨0596:CRMIMD⟩2.3.CO;2

Salarashayeri, A. F., & Siosemarde, M. (2012). Prediction of Soil Hydraulic Conductivity from Particle-Size Distribution.
doi: 10.5281/zenodo.1055779

Schrank, I., Löder, M. G. J., Imhof, H. K., Moses, S. R., Heß, M., Schwaiger, J., & Laforsch, C. (2022). Riverine microplastic contamination in southwest Germany: A large-scale survey. , *10*, 794250. Retrieved from https://www.frontiersin.org/articles/10.3389/feart.2022.794250/full doi: 10.3389/feart.2022.794250

Van Genuchten, M. T. (1981). Analytical solutions for chemical transport with simultaneous adsorption, zero-order production and first-order decay. *Journal of Hydrology*, *49*(3-4), 213–233. doi: 10.1016/0022-1694(81)90214-6

Van Melkebeke, M., Janssen, C., & De Meester, S. (2020). Characteristics and Sinking Behavior of Typical Microplastics Including the Potential Effect of Biofouling: Implications for Remediation. *Environmental Science & Technology*, *54*(14), 8668–8680. doi: 10.1021/acs.est.9b07378

Woodward, J., Li, J., Rothwell, J., & Hurley, R. (2021). Acute riverine microplastic contamination due to avoidable releases of untreated wastewater. *Nature Sustainability*, *4*(9), 793–802. doi: 10.1038/s41893-021-00718-2

Yalin, M. S. (1964). Geometrical Properties of Sand Wave. *Journal of the Hydraulics Division*, *90*(5), 105–119. doi: 10.1061/JYCEAJ.0001097

Yuan, Z., Nag, R., & Cummins, E. (2022). Human health concerns regarding microplastics in the aquatic environment - from marine to food systems. *Science of The Total Environment*, *823*, 153730. doi: 10.1016/J.SCITOTENV.2022.153730

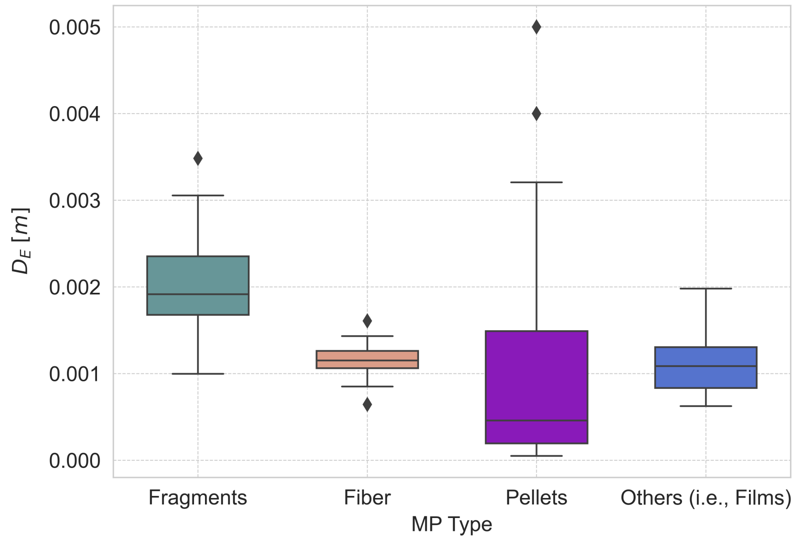

Figure S1. Scatter plot displaying the relationship between the grain shape factor (E) and the Corey shape factor (csf). The fitted power-law curve is represented by a black line, with the equation $E = 1.149csf^{-0.097}$ indicating a moderate negative correlation. The coefficient of determination, R^2, is 0.599. Data extracted from Van Melkebeke et al. (2020) and Le Roux (2004).

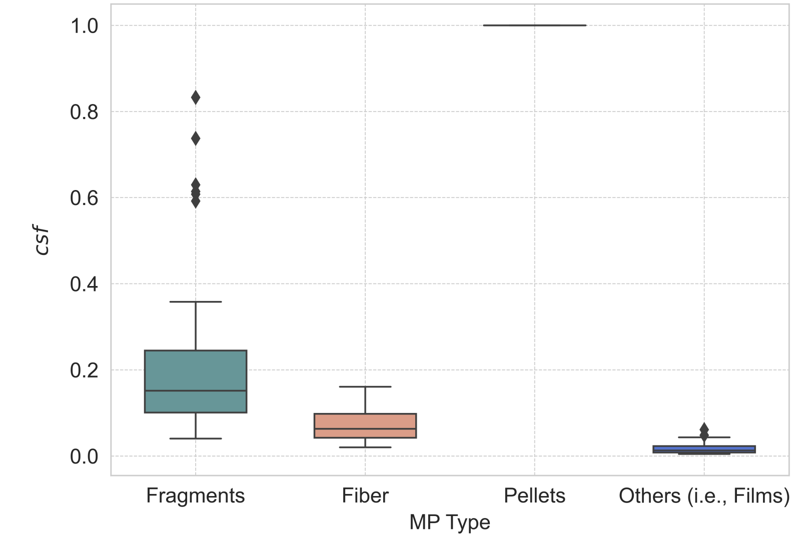

Figure S2. Boxplot displaying the Corey Shape Factor (CSF) for different types of MP particles. All pellets have a CSF of 1 due to their perfectly round shape, which results in the collapsed boxplot representation, whereas fragments and fibers exhibit a wider variation shape (Van Melkebeke et al., 2020; Le Roux, 2004).

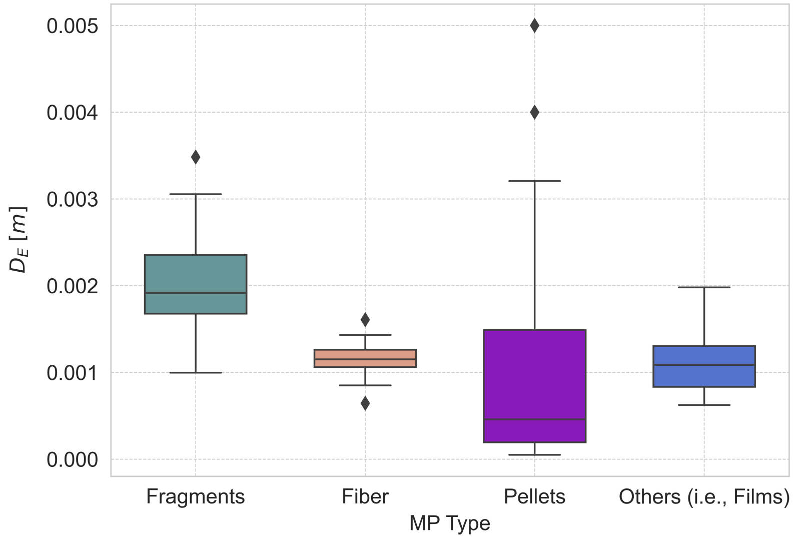

Figure S3. Boxplot of the equivalent diameters (D_n) for various MP types. The plot shows a comparison of the median, range, and outliers for the sizes of fragments, fibers, pellets, and other MP forms, such as films (Van Melkebeke et al., 2020; Le Roux, 2004).

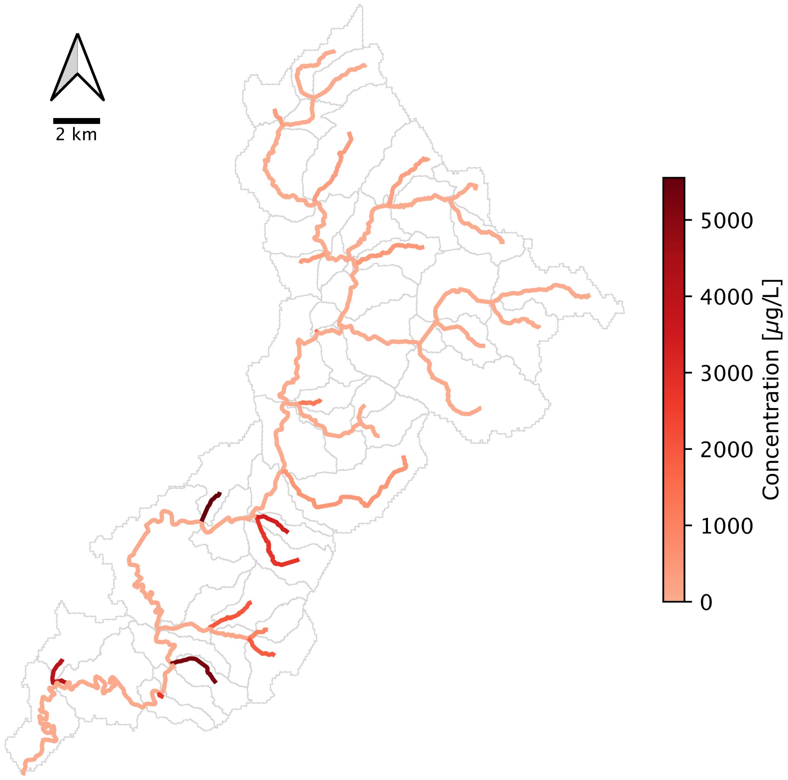

Figure S4. Modeled spatial distribution of microplastic (MP) concentrations [µg/L] across the river network for Scenario 1 (High Fibers).

Table S1. Summary of Transient Storage Time (τ), Reynolds Number (Re), and Characteristic Length (L), data extracted from Jackson (2010)

	τ $[s]$	Re $[-]$	L_c $[m]$		τ $[s]$	Re $[-]$	L_c $[m]$
Lab: Pool&Riffle	324	3.40E+04	8.5	River	1200	2.20E+06	95
Lab: Pool&Riffle	220	4.40E+04	8.5	River	900	1.20E+06	100
Lab: Pool&Riffle	208	5.00E+04	8.5	River	1860	6.70E+06	125
Streams	37	3.60E+04	1.5	River	1890	3.80E+06	125
Streams	56	4.90E+04	2.5	River	1770	3.60E+06	125
Streams	95	3.90E+04	2	River	2190	6.30E+06	70
Streams	29	2.70E+04	1	River	1830	4.30E+06	70
Streams	76	3.40E+04	1.9	River	1380	2.80E+06	70
Streams	100	9.90E+03	1.5	River	2220	6.30E+06	70
Streams	107	4.90E+04	2	River	1200	2.50E+06	85
Streams	56	6.30E+04	2.1	River	2820	6.70E+06	100
Streams	55	4.60E+04	2.1	River	3210	4.30E+06	111
Streams	71	4.60E+04	2.3	River	2700	7.50E+06	250
Streams	77	3.60E+04	3.1	River	2500	8.80E+06	250
Streams	48	3.10E+05	3.2	River	4000	1.00E+07	250
Streams	93	2.50E+05	3.9				
Streams	70	2.60E+05	3.1				
Streams	206	3.50E+04	2.4				
Streams	162	1.40E+05	2.4				

Table S2. Average proportions of microplastic types (fibers, pellets, fragments, others) across selected studies. Only country names are shown.

**Study**	**Country**	**Fibers**	**Pellets**	**Fragment**	**Others**	**Total [%]**	**Scenario**
Akdogan et al., (2023)	Turkey	88.00	2.00	8.00	2.00	100.00	
Liu et al., (2023)	China	72.10	0.00	23.40	4.60	100.10	
Yuan et al., (2022)	China	62.01	4.54	15.59	17.86	100.00	
Han et al., (2020)	China	93.12	4.74	2.14	0.00	100.00	
Mani et al., (2015)	Germany	2.50	58.40	37.50	1.10	99.50	3
Woodward et al., (2021)	UK	97.59	1.21	0.80	0.40	100.00	1
Margenant et al., (2021)	Spain	0.70	4.00	95.30	0.00	100.00	2
Schrank et al., (2022)	Germany	6.50	1.72	90.70	1.00	99.92	
Kunz et al., (2023)	Taiwan		1.10	96.63	2.19	99.92	
**Average**		52.82	9.58	34.18	3.37	99.94	4

Sources: Akdogan et al. (2023); Liu et al. (2023); Yuan et al. (2022); Han et al. (2020); Mani et al. (2015); Woodward et al. (2021); Margenat et al. (2022); Schrank et al. (2022); Kunz et al. (2023)
